# Matrikines are key regulators in modulating the amplitude of lung inflammation in acute pulmonary infection

**DOI:** 10.1038/ncomms9423

**Published:** 2015-09-24

**Authors:** Samia Akthar, Dhiren F. Patel, Rebecca C. Beale, Teresa Peiró, Xin Xu, Amit Gaggar, Patricia L. Jackson, J. Edwin Blalock, Clare M. Lloyd, Robert J. Snelgrove

**Affiliations:** 1 Leukocyte Biology Section, National Heart and Lung Institute, Imperial College London, London SW7 2AZ, UK; 2 Division of Pulmonary, Allergy and Critical Care Medicine, University of Alabama at Birmingham and Lung Health Center, Department of Medicine, University of Alabama at Birmingham, Birmingham, AL 3529, USA; 3 Gregory Fleming James Cystic Fibrosis Center and Program in Protease and Matrix Biology, University of Alabama at Birmingham, Birmingham, Alabama 35294, USA; 4 Birmingham V.A. Medical Center, Birmingham, Alabama 35294, USA

## Abstract

Bioactive matrix fragments (matrikines) have been identified in a myriad of disorders, but their impact on the evolution of airway inflammation has not been demonstrated. We recently described a pathway where the matrikine and neutrophil chemoattractant proline–glycine–proline (PGP) could be degraded by the enzyme leukotriene A_4_ hydrolase (LTA_4_H). LTA_4_H classically functions in the generation of pro-inflammatory leukotriene B_4_, thus LTA_4_H exhibits opposing pro- and anti-inflammatory activities. The physiological significance of this secondary anti-inflammatory activity remains unknown. Here we show, using readily resolving pulmonary inflammation models, that loss of this secondary activity leads to more pronounced and sustained inflammation and illness owing to PGP accumulation. PGP elicits an exacerbated neutrophilic inflammation and protease imbalance that further degrades the extracellular matrix, generating fragments that perpetuate inflammation. This highlights a critical role for the secondary anti-inflammatory activity of LTA_4_H and thus has consequences for the generation of global LTA_4_H inhibitors currently being developed.

## Abstract (web summary)

Proteases degrade extracellular matrix during inflammation, releasing peptides that can recruit neutrophils. Here the authors show that degradation of such bioactive peptide by the enzyme leukotriene A4 hydrolase is critical to limit pulmonary inflammation during bacterial infection in mice.

## Graphical abstract

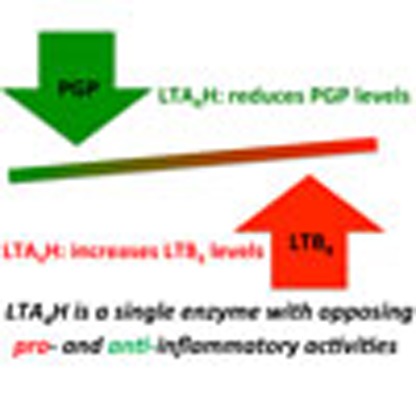

Neutrophils are critical components of the body’s immune response, being readily mobilized to the site of infection and destroying invading micro-organisms^1^,^2^. Recruitment of neutrophils into the tissue in response to infection is mediated by an array of chemoattractant signals, including lipid molecules such as leukotriene B_4_ (LTB_4_) and the glutamic acid–leucine–arginine^+^ (ELR^+^) class of chemokines. ELR^+^ chemokines, including interleukin (IL)-8 in humans and KC and MIP-2 in mice, exert their activity by binding to receptors CXCR1/2 (ref. 3). Despite their clear antimicrobial capacity, neutrophils are not indiscriminate killers but their inflammation is tightly regulated and they are able to elegantly and specifically shape many facets of the elicited immune response^4^,^5^. However, owing to their potent arsenal, an over-exuberant or persistent neutrophilic inflammation is implicated in the pathologies of chronic diseases such as chronic obstructive pulmonary disease (COPD) and cystic fibrosis (CF)^6^,^7^.

A protease–antiprotease imbalance is a hallmark of many chronic lung diseases, with the presence and activity of several matrix metalloproteinases (MMPs) and neutrophil elastase (NE), in particular, correlating with COPD pathology. Such proteases target the extracellular matrix (ECM) for degradation, disrupting tissue architecture and releasing ECM-derived chemoattractant signals, termed ‘matrikines’, which can perpetuate inflammation^8^. Models of exposure to cigarette smoke show that both breakdown of ECM (by neutrophil or macrophage elastase) and accumulation of neutrophils are required for pathological changes^9^,^10^,^11^,^12^. It is plausible therefore that a protease-/matrikine-driven inflammation may underlie the pathology observed in such chronic diseases.

The tripeptide proline–glycine–proline (PGP) is one such matrikine, being a neutrophil chemoattractant derived from ECM collagen that exerts its activity by mimicking key sequences found in ELR^+^ chemokines and binding to CXCR1/2 (ref. 13). PGP is generated from collagen via the sequential enzymatic activity of MMPs (specifically MMP-8 and -9) and prolylendopeptidase (PE)^14^. Significant quantities of PGP are found in patients with chronic lung diseases such as COPD, CF and bronchiolitis obliterans syndrome^14^,^15^,^16^,^17^,^18^. Recently, we have identified a novel anti-inflammatory pathway whereby PGP is readily degraded by the extracellular activity of the enzyme leukotriene A_4_ hydrolase (LTA_4_H)^19^. We subsequently demonstrated that this LTA_4_H-mediated PGP degradation is perturbed by cigarette smoke, contributing to the accumulation of PGP in COPD^18^,^19^.

LTA_4_H is classically recognized for its intracellular epoxide hydrolase activity, whereby it generates the pro-inflammatory lipid mediator LTB_4_ (refs 20, 21). LTB_4_ may bind to receptors BLT1 or BLT2; while BLT1 is a high-affinity and specific receptor for LTB_4_, BLT2 binds LTB_4_ with substantially lower affinity and can also bind to other eicosanoids^22^. The physiological functions of LTB_4_ are attributed to signalling through BLT1. LTB_4_ can drive the recruitment and activation of an array of cells including neutrophils and is thus implicated in protection against invading micro-organisms but also in the pathology of an array of diseases^23^,^24^,^25^,^26^,^27^. Thus, LTA_4_H represents an unusual enzyme with opposing pro- and anti-inflammatory activities that dictate the amplitude and persistence of neutrophilic inflammation^28^. There has been significant interest from pharmaceutical companies to target LTA_4_H therapeutically to alleviate LTB_4_-mediated pathologies, but despite the generation of several excellent inhibitors, these drugs have failed to demonstrate clinical efficacy^29^,^30^. The lack of success of these compounds could feasibly be due to their failure to distinguish between the opposing roles of LTA_4_H and thus inadvertently prevent PGP degradation.

The critical role of LTA_4_H in generating LTB_4_ and the significance of this lipid mediator in multiple inflammatory settings are undeniable. While it is clear that LTA_4_H possesses a secondary anti-inflammatory role in degrading PGP, the relative physiological significance of this activity remains unclear. To address this, we have manipulated the LTA_4_H pathways in a murine model of *Haemophilus influenzae* b (Hib). *H. influenzae* is a Gram-negative coccobacillus, of which there are encapsulated and unencapsulated strains^31^,^32^. Unencapsulated strains are termed non-typeable (NTHI) and are frequent causes of exacerbations of COPD and asthma. Encapsulated strains are divided into serotypes, of which there are six (a–f), based on their capsular antigen. Serotype B (Hib) is the cause of most invasive *H. influenzae* infections, which include pneumonia, meningitis, septicaemia and epiglottitis^33^. However, in most people Hib is found as a commensal of the nasopharynx, with the bacteria becoming invasive in only a small number of cases.

The murine model of Hib infection was chosen to dissect the dual activities of LTA_4_H, as it represents a non-complicated infection whereby the pathogen is readily cleared but elicits a robust pulmonary neutrophilia that is rapidly resolved. In this setting, it is feasible to address whether a failure to degrade PGP can lead to an augmented and prolonged neutrophilia to what is a relatively innocuous pulmonary insult. It thus provides an ideal model to probe the relative roles of LTB_4_ and PGP in protection and pathology. In this context, LTB_4_ was shown to be critical to bacterial phagocytosis and killing on a per-cell basis, but did not control the magnitude of the inflammatory response or illness. Conversely, a failure of LTA_4_H-mediated degradation of PGP led to accumulation of the peptide and a marked augmentation in inflammation and illness. This manifested primarily as a PGP-mediated increase in neutrophil numbers in the lung and airways, and a secondary protease imbalance that resulted in generation of elastin-derived matrikines that promoted a secondary macrophage infiltrate. Thus the failure to degrade PGP can have significant deleterious consequences, highlighting the key physiological role of the secondary anti-inflammatory activity of LTA_4_H and the prominent pathological role of PGP in inducing a general protease–matrikine imbalance.

## Results

### Dual-LTA_4_H activities are functional during Hib infection

**Figure 1:**
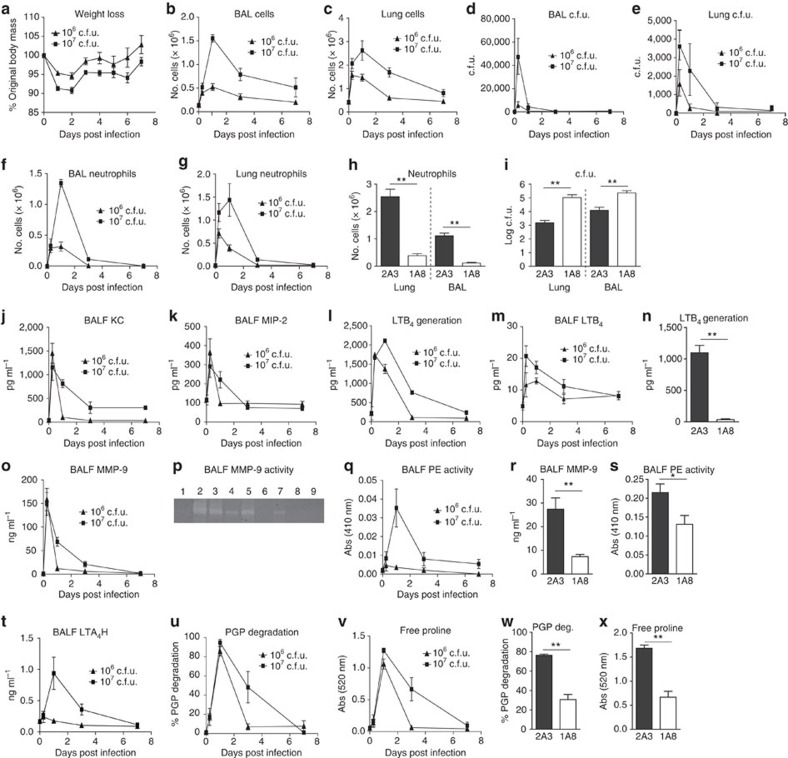
Dual-LTA_4_H activities are functional during Hib infection. 129/S6 mice were infected with Hib and weight loss assessed daily (**a**). Total cell numbers in the airways (**b**) and lung tissue (**c**) of Hib-infected mice were measured. Bacterial burden was assessed by performing serial dilutions of BALF (**d**) and lung homogenate (**e**) on Brain Heart Infusion agar. The number of neutrophils recruited into the airways (**f**) and lung tissue (**g**) of Hib-infected mice was determined by flow cytometry. Mice infected with 1 × 10^7^ Hib were administered control (2A3) or neutrophil-depleting (1A8) antibody, and neutrophil numbers (**h**) and c.f.u. (**i**) were assessed at 24 h post infection. The concentration of KC (**j**) and MIP-2 (**k**) in the BALF was determined by ELISA. Intracellular epoxide hydrolase activity of BAL cells (**l**). LTB_4_ levels in BALF of Hib-infected mice (**m**). Mice infected with 1 × 10^7^ Hib were administered 2A3 or 1A8 antibody and intracellular epoxide hydrolase activity of BAL cells determined at 24 h post infection (**n**). Amounts of total MMP-9 (**o**) in BALF were assessed by ELISA and MMP-9 gelatinolytic activity (**p**) was assessed by gelatin zymography (representative image depicted: lane 1=naive; 2=1 × 10^6^ 6 h; 3=1 × 10^7^ 6 h; 4=1 × 10^6^ 24 h; 5=1 × 10^7^ 24 h; 6=1 × 10^6^ day 3; 7=1 × 10^7^ day 3; 8=1 × 10^6^ day 7; 9=1 × 10^7^ day 7). (**q**) PE activity in BALF at different times after Hib infection. Mice infected with 1 × 10^7^ Hib were administered 2A3 or 1A8 antibody, and total MMP-9 levels (**r**) and PE activity (**s**) in the BALF were determined at 24 h post infection. Total LTA_4_H levels in BALF were assessed by ELISA at different times post infection (**t**). BALF, from different time points post Hib infection, was incubated with PGP and degradation was assessed after 2 h by mass spectrometry (**u**) or release of free proline (**v**). Mice infected with 1 × 10^7^ Hib were administered 2A3 or 1A8 antibody and PGP degradation by BALF at 24 h was assessed by mass spectrometry (**w**) or release of free proline (**x**). Data (mean±s.e.m.) are representative of at least two experiments with 5–6 mice per group.*P<0.05; **P<0.01 using the Mann–Whitney test.

Infection of 129/S6 mice with Hib elicited a transient, mild weight loss peaking at 24–48 h post infection, which correlated with the size of the inoculating dose of bacteria (Fig. 1a). Coinciding with peak illness was a robust, readily resolving inflammation observed in the airways (Fig. 1b) and lungs (Fig. 1c). Consequently, bacteria were promptly cleared from the airways (Fig. 1d) and lung parenchyma (Fig. 1e) of infected mice. The pulmonary infiltrate was predominantly neutrophilic, with Hib inducing a rapid, dose-dependent, influx of neutrophils peaking at 24 h post infection (Fig. 1f,g). Secondary to this neutrophilic inflammation was a relatively modest infiltration of monocytes/macrophages, natural killer cells and T cells (Supplementary Fig. 1A–E, respectively). Within this system, alveolar macrophages (Supplementary Fig. 1F–H) and neutrophils (Fig. 1h,i) were critical to the clearance of Hib, as their depletion significantly compromised bacterial clearance.

The arrival of neutrophils into the lungs and airways of Hib-infected mice was preceded by the release of ELR^+^ CXC chemokines KC (Fig. 1j) and MIP-2 (Fig. 1k) into the bronchoalveolar lavage fluid (BALF). Furthermore, conventional intracellular epoxide hydrolase activity of LTA_4_H was augmented by infection (Fig. 1l) and the neutrophil chemoattractant LTB_4_ was released into the BALF (Fig. 1m). This elevation in epoxide hydrolase activity of LTA_4_H was seemingly attributable to the substantial neutrophilic infiltrate, with the activity markedly reduced following neutrophil depletion (Fig. 1n). Key PGP-generating enzymes MMP-9 (Fig. 1o,p) and PE (Fig. 1q) were induced by Hib infection, but PGP was undetectable at any time point. Neutrophils are a prominent source of these PGP-generating enzymes enabling a feed-forward vicious circle of neutrophilic inflammation^14^,^19^,^34^,^35^,^36^. Accordingly, neutrophil depletion in Hib-infected mice resulted in a significant reduction in BALF MMP-9 levels (Fig. 1r) and PE activity (Fig. 1s). The failure to detect PGP, despite the presence of MMP-9/PE, could be attributable to extracellular release of LTA_4_H (Fig. 1t) and its capacity to degrade PGP (Fig. 1u,v). As previously postulated^19^, neutrophils are likely a source of extracellular LTA_4_H, as their depletion resulted in reduced capacity of BALF to degrade PGP (Fig. 1w,x). Thus both the pro-inflammatory LTB_4_-generating and the anti-inflammatory PGP-degrading activities of LTA_4_H are operational within this acute pulmonary neutrophilia model. The nature of this elicited response to Hib observed in 129/S6 mice was comparable in Balb/c and C57BL/6 mice (Supplementary Fig. 2).

### Loss of LTB_4_ signalling compromises Hib clearance

**Figure 2:**
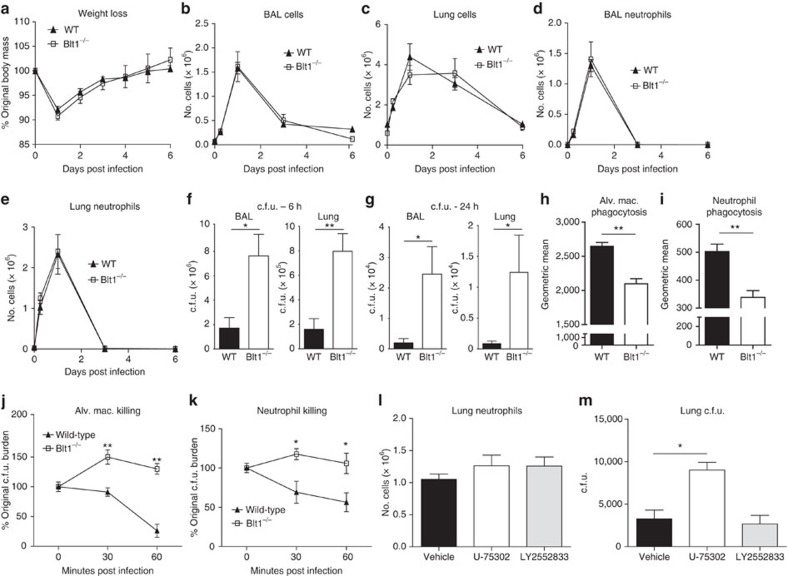
An absence of LTB_4_ signalling does not alter pulmonary inflammation but compromises bacterial clearance. Blt1−/− mice and WT controls were infected intranasally with 1 × 10^7^ Hib and weight loss was assessed daily and expressed as a percentage of the original body mass (**a**). Total cell numbers in the airways (**b**) and lung tissue (**c**) of Hib-infected mice were enumerated. The number of neutrophils recruited into the airways (**d**) and lung tissue (**e**) of Hib-infected mice was determined by flow cytometry. Bacterial burden was assessed by performing serial dilutions of BALF and lung homogenate, at 6 h (**f**) and 24 h (**g**), on Brain Heart Infusion (BHI) agar plates. Phagocytosis of pHrodo-conjugated Hib by alveolar macrophages (**h**) and neutrophils (**i**) was assessed by flow cytometry. The capacity of WT and blt1−/− alveolar macrophages (**j**) and neutrophils (**k**) to kill Hib was assessed. Hib-infected mice were administered vehicle (PBS), BLT1 antagonist (U-75302) or BLT2 antagonist (LY2552833), and lung neutrophil numbers (**l**) and c.f.u. (**m**) were assessed at 24 h post infection. Data (mean±s.e.m.) are representative of at least two experiments with 5–6 mice per group. *P<0.05; **P<0.01 using the Mann–Whitney test.

Physiological functions attributed to LTB_4_ are relayed through receptor BLT1. To infer the role of LTB_4_ in our Hib model, wild-type (WT) and blt1−/− mice were infected with 1 × 10^7^ colony-forming unit (c.f.u.) of Hib. WT and blt1−/− mice displayed comparable weight loss (Fig. 2a) and inflammation into the airways (Fig. 2b) and lung parenchyma (Fig. 2c) in response to Hib. Specifically, neutrophilic inflammation into the airways (Fig. 2d) and lung tissue (Fig. 2e) was indistinguishable between Hib-infected WT and blt1−/− mice. Despite comparable cellular inflammation, clearance of Hib was compromised in blt1−/− mice at 6 (Fig. 2f) and 24 h (Fig. 2g) post infection. A plethora of studies have highlighted the capacity of LTB_4_ to augment the phagocytic and antimicrobial potential of macrophages and neutrophils on a per-cell basis^26^,^37^,^38^,^39^,^40^,^41^,^42^,^43^,^44^,^45^,^46^,^47^,^48^,^49^,^50^. Alveolar macrophages (Fig. 2h) and neutrophils (Fig. 2i) from blt1−/− mice exhibited a significant reduction in their capacity to phagocytose pHrodo-labelled Hib relative to WT controls. Furthermore, blt1−/− alveolar macrophages (Fig. 2j) and neutrophils (Fig. 2k) exhibited reduced bacterial killing of Hib on a per-cell basis relative to WT counterparts.

The comparable cellular inflammation but compromised bacterial clearance observed at 24 h post infection in Hib-infected blt1−/− mice relative to WT controls was recapitulated through administration of a BLT-1-specific antagonist U-75302 to Hib-infected WT mice (Fig. 2l,m). Administration of a preferential BLT2 antagonist LY2552833 to Hib-infected WT mice had no effect on cellular inflammation or bacterial clearance (Fig. 2l,m), suggesting that BLT2 is not important in regulating these parameters within this model.

The enzyme 5-lipoxygenase (5-LO) generates leukotriene A_4_ (LTA_4_), which is subsequently hydrolysed by LTA_4_H into LTB_4_ (Supplementary Fig. 3A). To further verify the role of LTB_4_ in the outcome of Hib infection, we administered the 5-LO inhibitor zileuton to infected animals. While zileuton will inhibit LTB_4_ formation, there is the caveat that it could also disrupt production of cysteinyl leukotrienes (LTC_4_, D_4_, E_4_) and lipoxins since they share a common LTA_4_ intermediate (Supplementary Fig. 3A). Zileuton-administered Hib-infected mice showed comparable cellular inflammation into the airways and lungs at 24 h (Supplementary Fig. 3B,C) relative to control-treated animals. Furthermore, zileuton administration did not alter neutrophil numbers (Supplementary Fig. 3D,E) but did compromise bacterial clearance (Supplementary Fig. 3F,G).

### Hib-infected lta4h−/− mice display augmented inflammation

**Figure 3:**
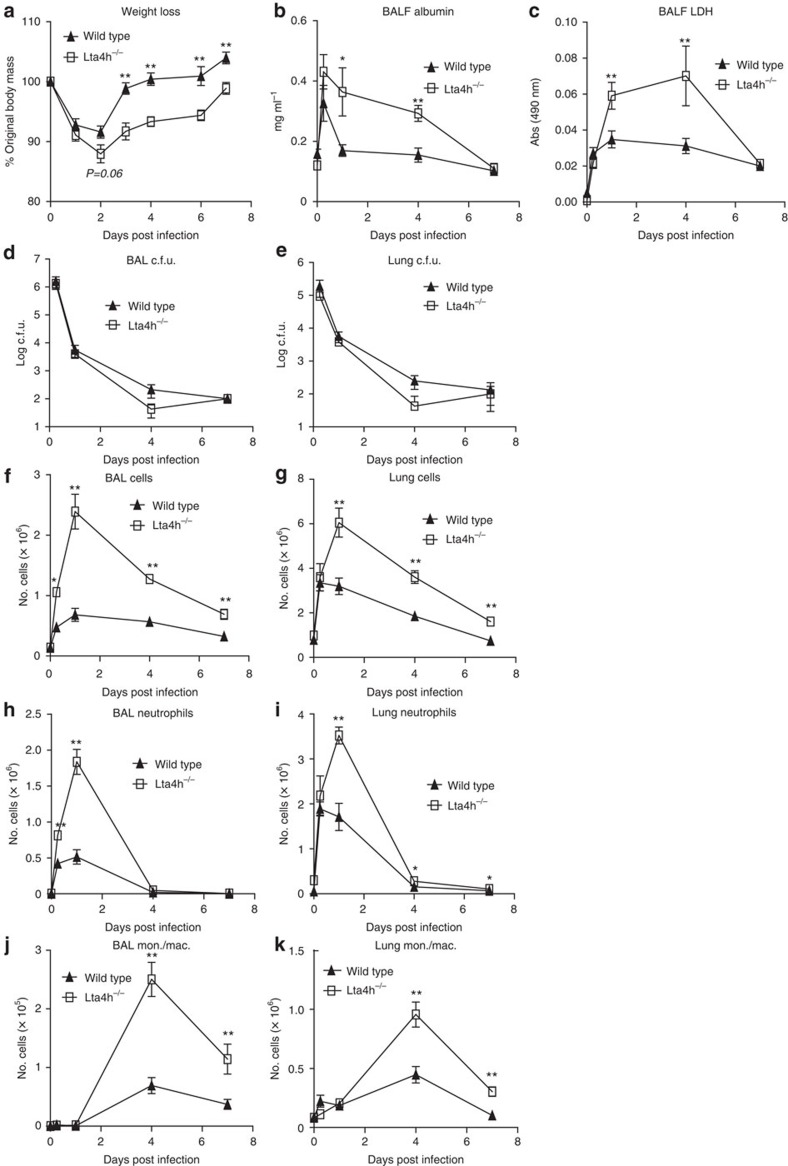
Hib-infected lta4h−/− mice display augmented illness and pulmonary inflammation. Lta4h−/− mice and littermate controls were infected intranasally with 1 × 10^7^ Hib, and weight loss was assessed daily and expressed as a percentage of the original body mass (**a**). Levels of albumin (**b**) and lactate dehydrogenase (LDH; **c**) in the BALF were assessed by ELISA and an enzymatic assay, respectively. Bacterial burden was assessed by performing serial dilutions of BALF (**d**) and lung homogenate (**e**) on Brain Heart Infusion (BHI) agar plates. Total cell numbers in the airways (**f**) and lung tissue (**g**) of Hib-infected mice was enumerated. The number of neutrophils recruited into the airways (**h**) and lung tissue (**i**) of Hib-infected mice was determined by flow cytometry. The number of infiltrating monocytes/macrophages recruited into the airways (**j**) and lung tissue (**k**) of Hib-infected mice was determined by flow cytometry. Data (mean±s.e.m.) are combined from two separate experiments with 4–5 mice per group and are representative of 3–4 experiments.*P<0.05; **P<0.01 using the Mann–Whitney test.

Having inferred the role of LTB_4_ in Hib infection, it was now of interest to determine the phenotype of Hib-infected lta4h−/− mice that lack not only the classical pro-inflammatory LTB_4_-generating activity but also the anti-inflammatory PGP-degrading activity. WT and lta4h−/− mice were infected with 1 × 10^7^ c.f.u. of Hib and ensuing illness and inflammation assessed. Hib-infected lta4h−/− mice exhibited a more pronounced weight loss that was slower to resolve relative to WT controls (Fig. 3a). In keeping with a more pronounced illness in lta4h−/− mice, they also displayed augmented pulmonary oedema (Fig. 3b) and cellular damage (Fig. 3c). The more pronounced illness observed in Hib-infected lta4h−/− mice relative to WT controls was not attributable to compromised bacterial clearance, as c.f.u. were comparable in BALF (Fig. 3d) and lung parenchyma (Fig. 3e). Instead, Hib-infected lta4h−/− mice showed a marked increase in cellular infiltrate into their airways (Fig. 3f) and lung tissue (Fig. 3g) relative to the WT animals, which was largely attributable to a substantial increase in neutrophils (Fig. 3h,i). Secondary to the augmented neutrophilia seen in lta4h−/− mice was an elevated infiltration of monocytes/macrophages into the airways (Fig. 3j) and lung tissue (Fig. 3k) relative to WT controls.

### PGP drives an exacerbated neutrophilic inflammation to Hib

**Figure 4:**
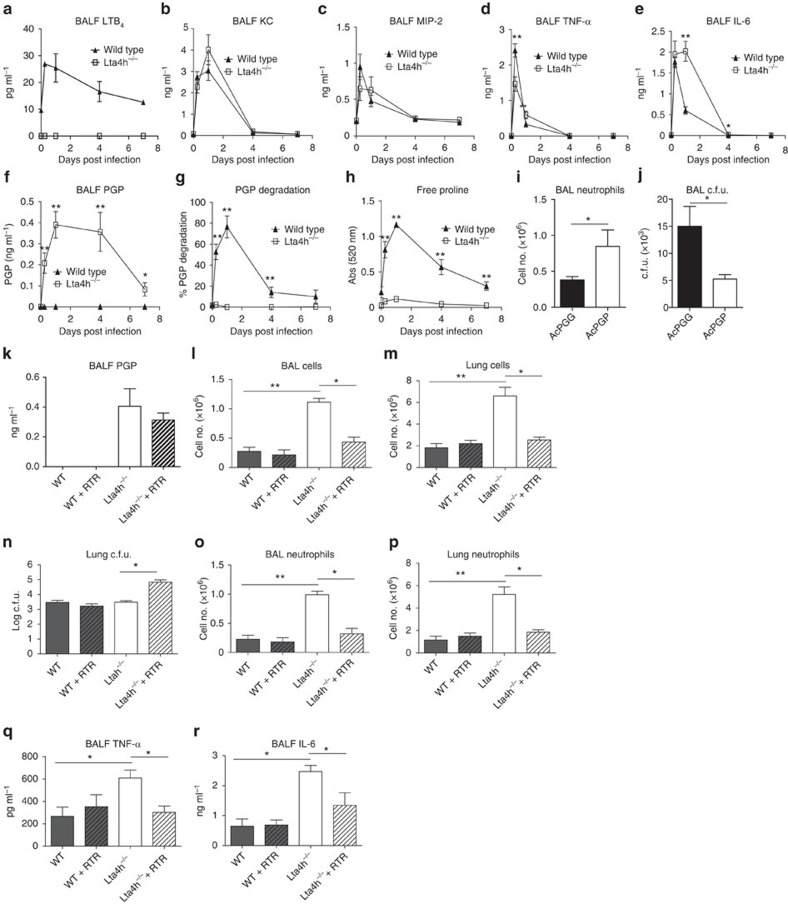
Failure to degrade PGP drives an exacerbated neutrophilic inflammation in response to Hib infection. Lta4h−/− mice and littermate controls were infected intranasally with 1 × 10^7^ Hib and the concentration of LTB_4_ (**a**), KC (**b**), MIP-2 (**c**), TNF-α (**d**) and IL-6 (**e**) in the BALF was determined. The concentration of PGP in BALF was determined by ESI–LC/MS/MS (**f**). BALF from different time points post Hib infection was incubated with PGP and degradation was assessed after 2 h by mass spectrometry (**g**) or release of free proline (**h**). Mice were infected intranasally with 1 × 10^7^ Hib and 2 h later treated intranasally with AcPGP or control peptide, AcPGG, and at 24 h post infection the number of neutrophils recruited into airways was determined by flow cytometry (**i**) and the bacterial burden in the BALF was assessed by performing serial dilutions on Brain Heart Infusion (BHI) agar plates (**j**). Lta4h−/− mice and littermate controls infected intranasally with 1 × 10^7^ Hib were administered vehicle or RTR peptide, and the concentration of PGP in BALF was determined by ESI–LC/MS/MS (**k**). Total cell numbers in the airways (**l**) and lung tissue (**m**) enumerated at 24 h post infection. Bacterial burden at this time point was assessed by performing serial dilutions of lung homogenate (**n**) on BHI agar plates. The number of neutrophils recruited into the airways (**o**) and lung tissue (**p**) of Hib-infected mice was determined by flow cytometry. The concentration of TNF-α (**q**) and IL-6 (**r**) in the BALF was determined by ELISA. Data (mean±s.e.m.) are combined from two separate experiments with 4–5 mice per group and are representative of 3–4 experiments (**a**–**h**) or are representative of at least two experiments with 5–6 mice per group (**i**–**r**). *P<0.05; **P<0.01 using the Mann–Whitney test.

The augmented neutrophilic inflammation observed in Hib-infected lta4h−/− mice, despite the absence of LTB_4_ (Fig. 4a), was not attributable to an elevation in KC and MIP-2 since levels in BALF (Fig. 4b,c) were comparable between WT and lta4h−/− mice. Other neutrophil chemoattractants C5a, lungkine/CXCL15 and ENA-78/CXCL5 were also comparable between WT and lta4h−/− mice (Supplementary Fig. 4A–C). Levels of pro-inflammatory cytokines tumour-necrosis factor (TNF)-α (Fig. 4d) and IL-6 (Fig. 4e), which can regulate neutrophil recruitment, were elevated at 24 h in Hib-infected lta4h−/− mice, but seemed insufficient alone to drive the observed phenotype. However, while no PGP was detectable in the BALF of Hib-infected WT mice, significant quantities were present in lta4h−/− animals (Fig. 4f). Accordingly, the capacity of BALF to degrade PGP was absent in Hib-infected lta4h−/− mice (Fig. 4g,h). PGP administration to Hib-infected WT mice augmented airway neutrophilia (Fig. 4i) and promoted bacterial clearance (Fig. 4j), supporting the notion that the phenotype of the lta4h−/− animals could be attributable to PGP accumulation.

To verify that the exacerbated illness and neutrophilic inflammation observed in lta4h−/− mice was due to PGP accumulation, we utilized a complementary peptide that specifically antagonizes PGP, namely arginine–threonine–arginine (RTR)^36^,^51^. Hib-infected WT and lta4h−/− mice were administered RTR, and inflammation and bacterial burden were assessed at 24 h post infection. While WT mice again displayed no PGP in their airways, control and RTR-treated lta4h−/− mice exhibited substantial quantities, highlighting that RTR does not reduce levels of PGP but rather binds and antagonizes it (Fig. 4k)^52^. Administration of RTR to Hib-infected lta4h−/− mice completely negated the augmented cellular infiltrate seen in the airways (Fig. 4l) and lungs (Fig. 4m) of these mice above that observed in the WT controls. Furthermore, administration of RTR to lta4h−/− mice compromised bacterial clearance (Fig. 4n)—presumably due to the loss of LTB_4_ and now the compensatory PGP that had previously conferred some augmented protection. In keeping with the role of PGP, the reduction in pulmonary inflammation observed in Hib-infected lta4h−/− mice administered RTR was due to a diminished neutrophilic infiltrate (Fig. 4o,p). The previously observed heightened TNF-α and IL-6 release in Hib-infected lta4h−/− mice (Fig. 4d,e) was normalized to WT levels following RTR treatment (Fig. 4q,r), suggestive that it is secondary to PGP. As a point of note, it has been suggested that loss of LTA_4_H function can lead to an accumulation of LTA_4_ and a subsequent lipoxin shunt, whereby levels of anti-inflammatory lipoxins are increased^53^. If this were the case in Hib-infected lta4h−/− mice, then one may envisage a reduced inflammation in response to the bacteria rather than the substantial PGP-driven increase observed. Furthermore, within our model system no lipoxins were detectable at any time point.

### Greater monocyte influx in Hib-infected lta4h−/− mice

PGP is a neutrophil chemoattractant and thus while it is rational that a greater neutrophil infiltrate is observed in lta4h−/− mice infected with Hib, it cannot explain the augmented ensuing infiltration of monocytes (Fig. 3j,k). Furthermore, there were comparable levels of classical monocyte-attracting chemokines preceding this monocytic infiltrate in lta4h−/− mice and littermate controls (Supplementary Fig. 4D–G). While collagen-derived PGP is not chemotactic for monocytes, elastin-derived matrikines are^11^,^54^,^55^. It was postulated that PGP accumulation in lta4h−/− mice may drive augmented neutrophilic inflammation with ensuing augmented elastase activity, elastin fragment generation and monocyte infiltration—in essence a protease/matrikine-driven inflammatory exacerbation.

**Figure 5:**
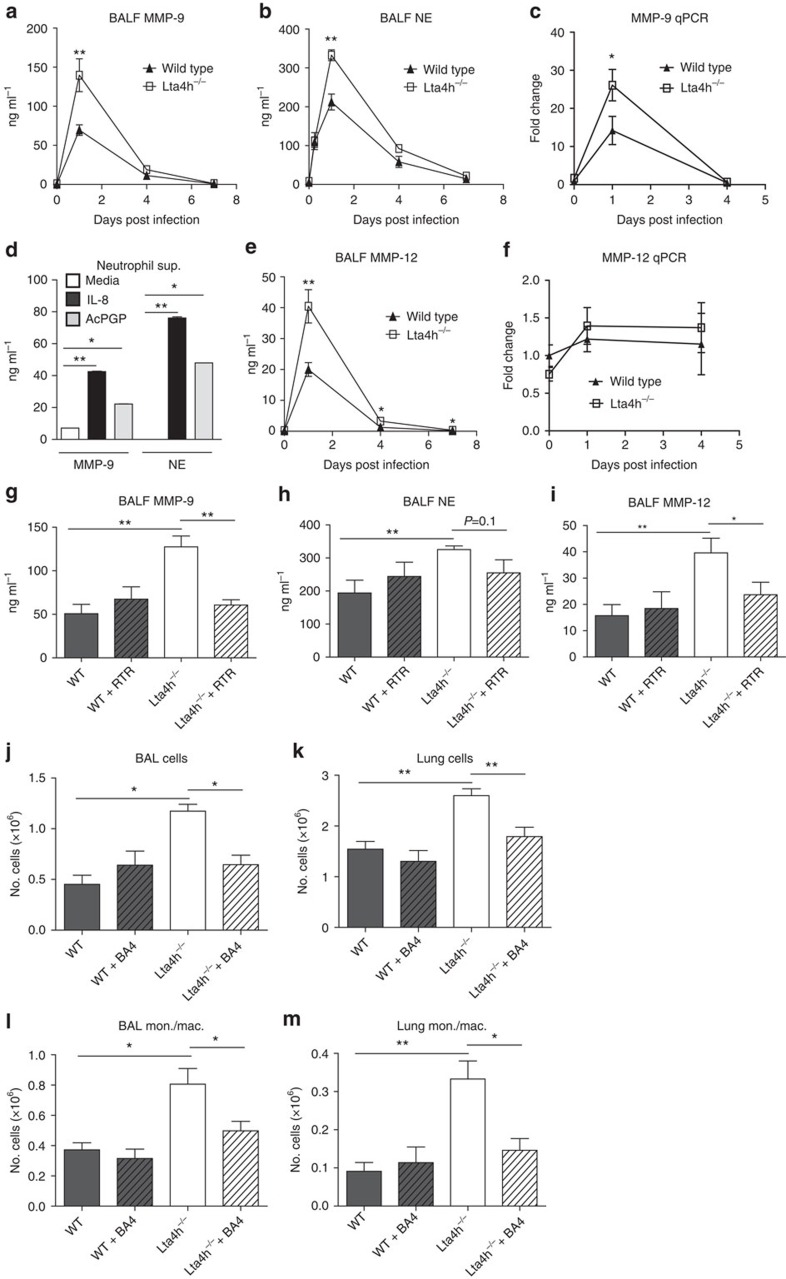
Augmented macrophage inflammation in Hib-infected lta4h−/− mice secondary to PGP accumulation and protease discord. Lta4h−/− mice and littermate controls were infected intranasally with 1 × 10^7^ Hib and levels of total MMP-9 (**a**) and NE (**b**) were assessed by ELISA in the BALF. Levels of MMP-9 mRNA were assessed in lung tissue by real-time PCR (**c**). Bone marrow neutrophils were incubated with media alone, IL-8 or AcPGP, and levels of MMP-9 and NE were assessed in the supernatant after 15 min (**d**). Levels of MMP-12 protein in the BALF were assessed by ELISA (**e**) and MMP-12 mRNA levels in lung tissue assessed by real-time PCR (**f**). Lta4h−/− mice and littermate controls infected intranasally with 1 × 10^7^ Hib were administered vehicle or RTR peptide, and the concentration of MMP-9 (**g**), NE (**h**) and MMP-12 (**i**) in the BALF was determined by ELISA at 24 h post infection. To determine the role of elastin-derived chemotactic fragments in driving cellular recruitment, lta4h−/− mice and littermate controls infected intranasally with 1 × 10^7^ Hib were administered control or BA4 (anti-elastin) antibody, and total cell numbers in the airways (**j**) and lung tissue (**k**) enumerated at 4 days post infection. In the same experiment, the numbers of infiltrating monocytes/macrophages in the airways (**l**) and lung tissue (**m**) was determined by flow cytometry at 4 days post infection. Data (mean±s.e.m.) are combined from two separate experiments with 4–5 mice per group and are representative of 3–4 experiments (**a**,**b**,**e**) or are representative of at least two experiments with 5–6 mice per group (**g**–**m**). Data (mean±s.d.) are representative of three experiments with at least triplicates (**d**). *P<0.05; **P<0.01 using the Mann–Whitney test.

In keeping with elevated neutrophilic inflammation in Hib-infected lta4h−/− mice, there was a significant increase in BALF levels of neutrophil-rich proteases MMP-9 (Fig. 5a) and NE (Fig. 5b). Furthermore, there was an augmentation in MMP-9 messenger RNA (mRNA) reflective of the substantial influx of neutrophils richly expressing this protease (Fig. 5c). Stimulation of bone marrow neutrophils with PGP elicited the release of MMP-9 and NE (Fig. 5d), demonstrating that the peptide was not only capable of recruiting neutrophils but also driving the secretion of their products. Intriguingly, Hib-infected lta4h−/− mice also displayed elevated macrophage elastase, MMP-12, levels in BALF (Fig. 5e). MMP-12 is predominantly expressed by alveolar macrophages and not neutrophils. There was no difference in MMP-12 mRNA levels between lta4h−/− mice and littermate controls (Fig. 5f), suggestive that increased levels in the BALF of knockout animals was not due to increased expression but rather release from alveolar macrophages.

In keeping with a blunted PGP-driven neutrophilic inflammation in RTR-treated lta4h−/− mice, there was a concomitant reduction in BALF MMP-9 (Fig. 5g) and NE levels were trending down (*P*=0.1; Fig. 5h). Furthermore, BALF levels of MMP-12 (Fig. 5i) were reduced, confirming that augmented levels in lta4h−/− animals were secondary to PGP accumulation. NE and MMP-12 can target elastin to generate fragments that are chemotactic for monocytes and could underlie their augmented numbers in Hib-infected lta4h−/− mice. To verify this, Hib-infected WT and lta4h−/− mice were treated with BA4 antibody (demonstrated to neutralize chemotactic elastin fragments), and cellular infiltrate assessed at day 4 post infection. BA4 administration to Hib-infected lta4h−/− mice blunted the elevated cellular infiltrate observed in airways (Fig. 5j) and lungs (Fig. 5k) of these animals and was largely attributable to a reduction in numbers of monocytes/macrophages (Fig. 5l,m).

### *S. pneumoniae* infection of lta4h−/− mice

To demonstrate that the exacerbated inflammation seen in Hib-infected lta4h−/− mice is not specific to a single model system, we investigated the dual roles of the enzyme following pulmonary infection with the Gram-positive bacteria *Streptococcus pneumoniae*. Infection of 129/S6 mice with *S. pneumoniae* elicited an early weight loss that correlated with size of the inoculating dose of bacteria (Supplementary Fig. 5A). However, unlike with Hib infection, bacterial burden is not efficiently eradicated from the airways or lung tissue (Supplementary Fig. 5B,C). Coinciding with the early weight loss was a robust inflammation in the airways (Supplementary Fig. 5D) and lung tissue (Supplementary Fig. 5E) that was again predominantly neutrophilic (Supplementary Fig. 5F,G). Key PGP-generating enzymes MMP-9 (Supplementary Fig. 5H) and PE (Supplementary Fig. 5I) were readily induced by *S. pneumoniae* infection, but PGP was undetectable at any time point by mass spectrometry. The failure to detect PGP, despite the presence of MMP-9/PE, was again attributable to extracellular release of LTA_4_H and its capacity to degrade PGP (Supplementary Fig. 5J).

WT and lta4h−/− mice were infected with either 1 × 10^5^ or 1 × 10^6^ c.f.u. of *S. pneumoniae*, and inflammation was assessed 24 h later. Lta4h−/− mice displayed a significant increase in cellular infiltrate into their airways (Supplementary Fig. 5K) with both doses of bacteria relative to littermate controls, which was largely attributable to a substantial increase in neutrophils (Supplementary Fig. 5L). The augmented neutrophilic infiltrate in *S. pneumoniae*-infected lta4h−/− mice relative to controls was not attributable to compromised bacterial clearance (Supplementary Fig. 5M) but rather to an accumulation of PGP (Supplementary Fig. 5N).

## Discussion

LTA_4_H is the rate-limiting enzyme in LTB_4_ biosynthesis, a potent pro-inflammatory mediator implicated in host defence but also an array of pathological conditions. However, we have recently identified a novel anti-inflammatory role for LTA_4_H in degrading the neutrophil chemoattractant PGP^19^. The physiological significance of this secondary PGP-degrading activity of LTA_4_H has not been addressed, and it is unknown whether a failure to degrade PGP can lead to adverse pathological sequelae, despite the absence of LTB_4_. To investigate the dual roles of LTA_4_H, we have primarily utilized an acute lung injury model of Hib infection—a model chosen since it evokes a robust pulmonary neutrophilia, but both inflammation and infection are readily resolved without complication. In this setting, it is feasible to assess the significance and risks of failing to degrade PGP and infer the importance of both LTA_4_H activities in protection and pathology.

Hib infection of WT mice resulted in an acute pulmonary neutrophilia that, together with resident alveolar macrophages, was important for the efficient clearance of bacteria. Concomitant with this early inflammation was induction of the classical intracellular epoxide hydrolase activity of LTA_4_H and release of LTB_4_, with neutrophils themselves seemingly the prominent source of the LTB_4_. Also concurrent with the pulmonary neutrophilic inflammation was an increase in levels of key PGP-generating enzymes MMP-9 and PE. Neutrophils are a prominent source of MMP-9 and PE and are thus capable of generating PGP and promoting their own recruitment and eliciting a vicious cycle of pathology if left unchecked^14^,^19^,^34^,^35^,^36^. However, despite the robust induction of MMP-9 and PE in response to Hib, no PGP was detectable at any time post infection. This was attributable to the extracellular release of LTA_4_H and its potent anti-inflammatory PGP-degrading capacity; thus both LTA_4_H activities are operational in our model system. We also report a failure to detect PGP, despite the presence of PGP-generating enzymes, in models of *S. pneumoniae* and influenza infection^19^. We would now argue that PGP degradation by LTA_4_H is the norm in settings of acute neutrophilia and instrumental in the efficient resolution of inflammation.

LTA_4_H is classically a cytosolic enzyme, but to perform its PGP-degrading activity it must be released to an extracellular environment. LTA_4_H is reported to exhibit wide tissue and cellular distribution^56^, which has been difficult to rationalize since leukocytes are the only cells to express both LTA_4_H and 5-LO required for leukotriene biosynthesis. It is feasible that the capacity of LTA_4_H to degrade PGP may account for the disproportionate distribution of LTA_4_H and 5-LO. We have previously demonstrated epithelial cells and neutrophils to be prominent sources of extracellular LTA_4_H^19^. Accordingly, we report a reduction in extracellular LTA_4_H in neutrophil-depleted Hib-infected mice. Thus neutrophils are capable of both initiating and resolving their own inflammation through the action of LTA_4_H, in disparate cellular locations.

Loss of LTB_4_ signalling through the use of blt1−/− mice or BLT1 antagonists, or administration of 5-LO inhibitor zileuton, did not alter pulmonary inflammation or illness in response to Hib infection, but significantly compromised bacterial clearance. The reduced capacity to control Hib infection in blt1−/− mice was attributable to reduced phagocytosis and killing of the bacteria on a per-cell basis by alveolar macrophages and neutrophils. A clear role for LTB_4_ in augmenting phagocytosis and microbicidal activity of macrophages and neutrophils is well established^26^,^37^,^38^,^39^,^40^,^41^,^42^,^43^,^44^,^45^,^46^,^47^,^48^,^49^,^50^, with the plethora of studies pertaining to Gram-negative bacteria, *Klebsiella pneumoniae*, particularly pertinent to our studies^26^,^37^,^39^,^44^,^45^.

Given the potent pro-inflammatory capacity of LTB_4_, it seems counterintuitive that there is not a reduced cellular infiltrate in blt1−/− mice or mice administered 5-LO inhibitor zileuton. However, it is interesting to note that reduced neutrophil recruitment to sites of inflammation in 5-LO−/− mice has been observed in some, but certainly not all, experimental models examined^27^,^57^. Indeed, a compromised pathogen clearance yet comparable cellular inflammation has been reported previously in mice with a disrupted LTB_4_ pathway^26^,^42^. Thus it may be that while LTB_4_ serves an important function in Hib killing on a per-cell basis, it is not critical for directing cellular inflammation within this model, potentially owing to redundancy with other neutrophil chemoattractants. Alternatively, it is feasible that an absence of LTB_4_ signalling reduces cellular inflammation and bacterial clearance, and this augmented TLR burden subsequently yields a greater release of conventional neutrophil chemokines that promote further neutrophil recruitment—thus masking the original deficit.

Lta4h−/− mice administered Hib displayed greater illness, which was attributable to an augmented cellular infiltrate and ensuing immunopathological sequelae. Most notable within this phenotype was the striking increase in neutrophilic inflammation of Hib-infected lta4h−/− mice, despite the loss of LTB_4_, and comparable levels of other classical neutrophil chemoattractants. This augmented neutrophilia was attributable to PGP accumulation in lta4h−/− mice, as neutralization of PGP completely ablated neutrophil infiltrate to WT levels. Levels of TNF-α and IL-6, which could regulate neutrophilic inflammation and illness, were also elevated in lta4h−/− mice, but were secondary to PGP accumulation. Thus we show that a failure to degrade PGP has serious implications in the control of neutrophilic inflammation, and that the anti-inflammatory activity of LTA_4_H has a clear physiological significance over and above that of its pro-inflammatory action in this setting.

It is perhaps surprising that, despite the clear role of LTB_4_ in protection against Hib infection, lta4h−/− mice (which lack LTB_4_) displayed a comparable bacterial burden to their littermate controls. However, while lta4h−/− mice lack LTB_4_, they possess an increase in PGP and ensuing neutrophilic inflammation that seemingly compensates *in vivo*. Neutrophils are critical effector cells within our Hib model and given the capacity of PGP to drive neutrophil recruitment and effector function, it is not surprising that PGP can prove protective against bacterial challenge, as highlighted by a previous study^58^. Indeed, we demonstrate that PGP administration to Hib-infected WT mice enhances neutrophilic recruitment and bacterial clearance. Furthermore, we clearly demonstrate that PGP is compensating for the loss of LTB_4_ and conferring protection against Hib challenge in lta4h−/− mice, as neutralization of the PGP with RTR restored the compromised bacterial clearance.

PGP can drive MMP-9 release from neutrophils and promote a feed-forward system that generates more PGP and further neutrophil recruitment^59^. Accordingly, we demonstrate that PGP can drive MMP-9 release from neutrophils and observe significantly greater MMP-9 levels in Hib-infected lta4h−/− mice—a phenotype that is lost if PGP is neutralized. In addition to MMP-9, we demonstrated Hib-infected lta4h−/− to exhibit a broader protease imbalance with an elevation in NE and MMP-12. The augmented NE is rational, given the elevated neutrophil numbers in lta4h−/− mice and the potential of PGP to drive NE from neutrophils. More surprising was the PGP-dependent increase in MMP-12 in Hib-infected lta4h−/− mice. Neutrophils do not produce MMP-12, and it is likely that alveolar macrophages represent the primary source in our Hib model. PGP is unable to directly elicit the release of MMP-12 from alveolar macrophages and one would predict that release results from a neutrophil-alveolar macrophage cross-talk. Secondary to the elevated neutrophilia and protease imbalance was an augmented monocytic/macrophage infiltrate in the lta4h−/− mice that was seemingly dependent on elastin-derived matrikines in these mice. While mature, cross-linked elastic fibres are not chemotactic for monocytes, cryptic sequences may be released upon proteolysis by elastolytic enzymes, such as MMP-12 or NE. Such sequences are hydrophobic penta- and hexapeptide repeats such as VGVAPG, which has been demonstrated to be chemoctactic for monocytes with optimal activity at ∼10 nM (refs 60, 61, 62). Furthermore, many chemotactic elastin fragments generated *in vivo* are also much larger, and thus the combined chemotactic potential of elastin-derived products towards monocytes could be substantial^11^. Therefore, the absence of LTA_4_H in response to a fairly innocuous challenge gives rise to an accumulation of PGP and neutrophils and a secondary accumulation of elastin-derived matrikines and macrophage numbers (Supplementary Fig. 6)—thus two distinct protease–matrikine systems are inherently linked and the implications of a failure to degrade PGP are significant.

Loss of LTA_4_H function clearly resulted in an exacerbated PGP-driven pulmonary inflammation and illness in response to Hib infection. However, the importance of the LTA_4_H PGP-degrading activity, even over and above the well-heralded LTB_4_-generating activity, is not unique to Hib infection, as we also report a comparable phenotype following challenge with *S. pneumoniae*. Previous interpretation of the role of LTA_4_H in neutrophilic inflammation had not considered the unrecognized PGP-degrading secondary activity of LTA_4_H and will indeed be complicated by the opposing roles of this bifunctional enzyme. While both 5-LO and LTA_4_H knockout mice exhibit reduced inflammation in models of ear inflammation and peritonitis relative to WT mice, the reduction is more pronounced in 5-LO knockouts^63^. It is feasible that deletion of LTA_4_H, while preventing LTB_4_ production, may promote PGP levels giving rise to the intermediary phenotype. Furthermore, in an elastase model of emphysema, lta4h−/− animals exhibited a blunted early neutrophilic response that gave way to elevated numbers at later time points—potentially pointing to disparate temporal roles for the two activities of LTA_4_H^64^. The significance of LTA_4_H and its dual opposing activities in different instances will be complicated by the availability of enzymes that generate LTA_4_ and PGP, the relative intra vs extracellular levels of LTA_4_H and many other confounding factors. It is feasible therefore that the relative importance of each of LTA_4_H’s pathways will be disease and even patient specific.

While we demonstrate the result of PGP accumulation in a system where it is normally degraded, it is worth considering the implications of these studies in the context of the chronic diseases where PGP is not efficiently degraded. PGP is elevated in lung diseases such as COPD and CF, often inversely correlating with disease severity^14^,^15^,^16^,^17^,^18^. The development of COPD is associated with an accumulation of neutrophils and macrophages and a protease imbalance (specifically MMPs and NE) leading to ECM attack^10^,^11^,^12^, and there is accumulating evidence that a protease–matrikine-mediated pathology is instrumental in the development of diseases^54^. We have previously demonstrated that cigarette smoke, a key risk factor in the development of COPD, can specifically inhibit the aminopeptidase activity of LTA_4_H^18^,^19^,^65^. In light of our current findings, it is likely that the failure of LTA_4_H to efficiently degrade PGP is an instrumental and potentially early step in this protease imbalance and augmented neutrophil and macrophage infiltrate. It is also noteworthy that *H. influenzae* and *S. pneumoniae* are common exacerbators of COPD, and that PGP and neutrophils/proteases have been demonstrated to spike with exacerbations^17^,^66^,^67^. One can thus envisage a scenario whereby such a bacterial challenge leads to further PGP generation in a setting, whereby the peptide cannot be degraded. Targeting the PGP pathway may therefore offer therapeutic potential in chronic neutrophilic lung diseases characterized by PGP accumulation, and in infectious exacerbations of these diseases^28^,^68^,^69^,^70^.

It is important to question the significance of these findings in the context of therapeutic strategies that seek to inhibit LTA_4_H to reduce LTB_4_-mediated pathologies^29^. There has been a significant pharmaceutical effort to generate LTA_4_H inhibitors, but despite the development of drugs that show excellent pharmacokinetic profiles, none of these compounds have shown efficacy in clinical trials. These inhibitors seem unlikely to distinguish between the opposing activities of LTA_4_H and may therefore inadvertently prevent PGP degradation with adverse effects.

In conclusion, we show for the first time the consequences of a failure to degrade PGP, highlighting a critical role for the secondary anti-inflammatory activity of LTA_4_H. In response to a relatively innocuous pulmonary challenge, accumulation of PGP leads to an augmented neutrophilic inflammation and ensuing illness, as well as a general protease imbalance that targets the ECM and further perpetuates inflammation. These studies have significant implications for our basic understanding of LTA_4_H biology, the cross-talk between protease–matrikine networks, the role of PGP in the pathology and development of chronic lung diseases and the design and application of LTA_4_H inhibitors by pharmaceutical companies.

## Methods

### *Haemophilus influenzae* b

Hib Eagan strain was a kind gift from Professor T. Hussell (Institute of Inflammation and Repair, The University of Manchester, UK). Bacteria were cultured at 37 °C in 5% CO_2_ in Brain Heart Infusion (BHI) broth (OXOID, Hampshire, UK) supplemented with 10 μg ml^−1^ of both Hemin (Roche, West Sussex, UK) and nictinamide adenine dinucleotide (Sigma-Aldrich, Dorset, UK) or on BHI agar (OXOID) supplemented with 4% Levinthals. Levinthals was made by adding 50% horse blood (TCS Biosciences, Buckingham, UK) to BHI broth and heating to 70 °C for 45 min. On cooling to 50 °C, 0.7 mg ml^−1^ nictinamide adenine dinucleotide was added and the supernatant was stored at −80 °C. Bacteria were cultured to an optical density (OD)_600_ of 0.3 (∼1 × 10^9^ c.f.u. ml^−1^) and stored at −80 °C in 10% glycerol as single-use aliquots.

### *S. pneumoniae*

*S. pneumoniae* (serotype 2) strain D39 (NCTC 7466, National Collection of Type Cultures, London, UK), a kind gift from Professor T. Hussell (Institute of Inflammation and Repair, The University of Manchester) was cultured at 37 °C in 5% CO_2_ on blood agar plates or in Todd-Hewitt broth (OXOID) supplemented with 0.5% yeast extract (OXOID) (THY broth) to an OD_600_ of 0.4 (∼1 × 10^8^ c.f.u. ml^−1^) and stored at −80 °C in 10% glycerol as single-use aliquots.

### Mice

Six- to eight-week-old female BALB/c and C57BL/6 mice were purchased from Harlan Olac Ltd (Oxon, UK). *Blt1*^*−/−*^, on a C57BL/6 background, were purchased from Jackson Labs (Bar Harbor, ME), and *Lta4h*^*−/−*^ mice and littermate controls were on a 129/S6 background and a gift from Dr Y.M. Shim (University of Virginia, Charlottesville, VA). All WT 129/S6 mice referred to within the manuscript are littermate controls of lta4h−/− mice. All mice were kept in specified pathogen-free conditions and provided autoclaved food, water and bedding. This study was carried out in accordance with the recommendations in the Guide for the Use of Laboratory Animals of Imperial College London. All animal procedures and care conformed strictly to the UK Home Office Guidelines under the Animals (Scientific Procedures) Act 1986, and the protocols were approved by the Home Office of Great Britain.

*Lta4h*^*−/−*^ and *Blt1*−/− mice were genotyped by PCR on genomic DNA extracted using the Extract-N-Amp Tissue PCR Kit (Sigma-Aldrich). The WT expression of LTA_4_H was detected by primers oIMR1720 (5′-CGAATCCATGCTTAAAATTGC-3′) and oIMR1721 (5′-GCGTTACGAACGTGAGACAA-3′) to yield a product size of 128 bp, and *Lta4h*^*−/−*^ LTA_4_H was detected by primers oIMR6916 (5′-CTTGGGTGGAGAGGCTATTC-3′) and oIMR6917 (5′-AGGTGAGATGACAGGAGATC-3′) to yield a product size of 280 bp. Amplification was achieved by PCR (94 °C for 3 min>35 × 2.5 min cycles (30 s at 94 °C>60 s at 60 °C>60 s at 72 °C)>72 °C for 2 min). The BLT1 expression was detected by primers oIMR8160 (5′-ATAGCTTTGTAGTGTGGAGCATCCTGA-3′), oIMR8161 (5′-TGGAAGACTTTATGCTCTTTGTTGGGA-3′) and oIMR8162 (5′-TGGATGTGGAATGTGTGCGAG-3′) to yield a product size of 433 bp for WT and 270 bp for mutant. Amplification was achieved by PCR (94 °C for 3 min>35 × 2.5 min cycles (30 s at 94 °C>60 s at 64 °C>60 s at 72 °C)>72 °C for 2 min). Amplification fragments were visualized on 2% agarose gels.

### Mouse infection model

Mice were anaesthetized with isoflourane and infected intranasally (i.n.) with 1 × 10^6^ or 1 × 10^7^ c.f.u. Hib or with 1 × 10^5^ or 1 × 10^6^ c.f.u. *S. pneumoniae* in 50 μl of sterile PBS. Mice were monitored daily and weight loss was recorded. In neutrophil depletion experiments, 129/S6 mice received intraperitoneal (i.p.) administration of 500 μg and i.n. administration of 200 μg of anti-Ly6G (1A8, Bio X Cell, West Lebanon, NH) or control rat IgG (2A3, Bio X Cell) the day preceding and the day of infection and culled 24 h later. In alveolar macrophage-depletion experiments, 129/S6 mice received i.n. administration of 50 μl of control or clodronate liposomes (ClodronateLiposomes.com) 24 and 48 h before infection and culled 24 h post infection. In some experiments, BLT-1-specific antagonist U-75302 (Cayman Chemical Company, Ann Arbor, MI) or BLT2-specific antagonist LY2552833 (Cayman Chemical Company) or vehicle controls were administered at a dose of 100 μg i.p. to 129/S6 mice 1 h before infection and 9 h post infection before being culled at 24 h post infection. In some experiments, mice were each i.p. administered 500 μg of zileuton (Sigma-Aldrich) the day preceding and the day of infection and culled 24 h later. Zileuton was dissolved in a 50% solution of dimethyl sulfoxide (DMSO) (Sigma-Aldrich) in saline, with a final volume of 100 μl. A similar volume of DMSO was given i.p. to control mice in these experiments. In some experiments, mice were administered i.n. 250 μg AcPGP (BACHEM, Bubendorf, Switzerland) or control AcPGG (BACHEM) peptide 2 h post infection and culled at 24 h. To neutralize PGP, mice were administered RTR peptide (Anaspec peptides, Fremont, CA) at a dose of 100 μg i.n. to mice 1 and 9 h post infection before being culled at 24 h. To neutralize elastin fragments, mice were administered BA4 (Sigma-Aldrich) or control antibody at a dose of 50 μg i.n. to mice on a daily basis from time of infection before being culled at day 4.

### Cell recovery and isolation

Mice were administered 3 mg pentobarbital and exsanguinated via the femoral artery. Serum was isolated by centrifugation for 8 min at 5,000*g* and frozen at –80 °C until required. The lungs were then inflated five times with 1.5 ml PBS via an intratracheal cannula and 100 μl from each mouse removed for enumeration of bacterial burden (see below). The remainder was centrifuged and the supernatant stored at –80 °C. The pellet was resuspended at 10^6^ cells per millilitre in R10F (RPMI containing 10% (vol/vol) FCS, penicillin (50 U ml^−1^), streptomycin (50 μg ml^−1^) and L-glutamine (2 mM)) and cell viability was assessed using trypan blue exclusion. Lung tissue was disrupted to a single-cell suspension by passage through a 100-μM sieve (BD labware, New Jersey). 100 μl from each mouse was again removed for enumeration of bacterial burden and the remaining cell suspension was centrifuged for 5 min at 800*g* and red blood cells lysed by resuspending pellets in ACK buffer (0.15 M ammonium chloride, 1 M potassium hydrogen carbonate and 0.01 mM EDTA, pH 7.2) for 3 min at room temperature before centrifugation (800*g* for 5 min) and resuspending in R10F. Cell viability was assessed by trypan blue exclusion and cells resuspended in R10F at 10^6^ cells per millilitre.

Alveolar macrophages were isolated by adherence of BAL fluid to plastic for 1 h in DMEM at 37 °C, 5% CO_2_ and shown to be >97% pure by flow cytometric analysis. To isolate bone marrow neutrophils, the femur and the tibia from both hind legs were removed and freed of soft tissue attachments, and the extreme distal tip of each extremity was cut off. HBSS containing 15 mM EDTA and 30 mM HEPES was forced through the bone with a syringe. After dispersing cell clumps and passage through a 100-μM sieve (BD labware), red blood cells were lysed by resuspending pellets in ACK buffer (for 3 min at room temperature) before centrifugation (800*g* 5 min) and washing with HBSS. The cell suspension was centrifuged (800*g*, 5 min, 4 °C) and resuspended in RPMI. The cells were layered onto a 72, 64, 52% Percoll gradient (Sigma-Aldrich) diluted in PBS (100% Percoll=nine parts Percoll and one part 10 × PBS), and centrifuged (1,500*g*, 30 min, room temperature) without braking. The neutrophils at the 64/72% interface were harvested and washed with 20 ml RPMI. Cell viability was assessed by trypan blue exclusion and confirmed to be >90% neutrophils by flow cytometry.

### Bacterial c.f.u. enumeration

BALF and lung suspension (as detailed above) were serially diluted in sterile PBS and plated onto appropriate agar plates (Columbia blood agar plates for *S. pneumoniae*, BHI agar with 4% Levinthals for Hib) and grown overnight at 37 °C with 5% CO_2_. Resultant colonies were counted manually. *S. pneumoniae* was confirmed by optochin sensitivity. Hib was confirmed by no growth on BHI agar without Levinthals.

### Flow cytometry

Single-cell suspensions were stained for surface markers in PBS containing 0.1% sodium azide and 1% BSA for 30 min at 4 °C and fixed with 2% paraformaldehyde. Data were acquired on a BD FACS Fortessa machine (BD Biosystems, UK). Forward-scatter and side-scatter gates were used to exclude debris, and dead cells were excluded using a fixable near-infrared dead cell stain kit for 633 or 635 nm excitation. Cell types were characterized by their forward- and side-scatter profiles and by their phenotypes, as depicted in Table 1.

### Treatment of neutrophils with AcPGP

Pre-warmed neutrophil suspensions (37 °C, 2 × 10^6^ cells per millilitre) were incubated in 1% BSA/HBSS with or without AcPGP (0.1 mg ml^−1^; BACHEM) or IL-8 (1 μg ml^−1^) for 15 min at 37 °C in 5% CO_2_. The level of enzymes released in cell-free supernatant was measured by enzyme-linked immunosorbent (ELISA)-based assays (see below).

### Alveolar macrophage and neutrophil phagocytsosis assay

Alveolar macrophages (pre-adhered; 1 × 10^5^) or bone-marrow-derived neutrophils (in suspension; 1 × 10^5^) from WT and blt1−/− mice were incubated with *H. influenzae* b (multiplicity of infection 20:1; with 10% serum) conjugated with pHrodo dye utilizing the pHrodo Phagocytosis Particle Labelling Kit (Invitrogen, USA) and phagocytosis assessed by flow cytometry according the manufacturer’s directions. pHrodo is a fluorogenic dye that exhibits low fluorescence at neutral pH but fluoresces in acidic environments, allowing accurate measurement of the engulfment of pHrodo-labelled bacteria into the acidic phagosome.

### Alveolar macrophage- and neutrophil-killing assay

Alveolar macrophages (pre-adhered; 5 × 10^5^) or bone-marrow-derived neutrophils (in suspension; 1 × 10^6^) from WT and blt1−/− mice were infected with *H. influenzae* b (multiplicity of infection 1:1) in RPMI with 5% serum at 37 °C. After 15 min, extracellular bacteria were removed by washing 3 × with ice-cold RPMI and cells were resuspended in 300 μl RPMI/5% serum and incubated at 37 °C. At 0, 30 and 60 min, 100 μl samples were removed and diluted into 900 μl H_2_O. Cells were allowed to lyse by standing at room temperature for 10 min and were subsequently vortexed vigorously to disperse bacteria. Samples were further diluted in H_2_O, plated on BHI agar with 4% Levinthals and grown overnight at 37 °C with 5% CO_2_. Resultant colonies were counted and % original c.f.u. burden determined relative to the *t*=0-min time point.

### Protease quantification and activity

The concentration of MMP-9 in BALF and neutrophil supernatants was measured using an ELISA, according to the manufacturer’s directions (R&D Systems, Minneapolis, MN). To assess MMP-9 gelatinolytic activity, BALF was mixed 1:1 (vol:vol) with Tris-Glycine-SDS Sample Buffer (Life Technologies Ltd, Paisley, UK) and loaded onto 10% zymogram (gelatin) gels (Life Technologies Ltd). Samples were subsequently electrophoresed at 125 V for 90 min in Tris-Glycine-SDS Running Buffer (Life Technologies Ltd). Following electrophoresis, gels were incubated in 1 × Zymogram Renaturing Buffer (Life Technologies Ltd) for 30 min at room temperatrue. To visualize the gelatinolytic activity, the gel was incubated in 1 × Zymogram Developing Buffer (Life Technologies Ltd) overnight at 37 °C before staining with SimplyBlue Safestain (Life Technologies Ltd).

The concentrations of LTA_4_H, NE and MMP-12 in BALF and/or neutrophil supernatants were measured using an ELISA, according to the manufacturer’s directions (USCN Life Science, Hubei, PRC).

### PE activity assay

Twenty microlitre of BALF was incubated with a specific substrate (2 mM Benzylcarboxy-Glycine-Proline-para-Nitroaniline, ZGP-pNa; Chem-Impex, Wood Dale, IL) at 37 °C and 5% CO_2_ and cleavage of para-nitroaniline (pNa) from the substrate by PE was detected using a spectrophotometer at 410 nm.

### ESI–LC/MS/MS for PGP detection

For peptide quantification in BALF, PGP and AcPGP were measured using a MDS Sciex (Applied Biosystems, Foster City, CA) API-4000 spectrometer equipped with a Shimadzu HPLC (Columbia, MD). For peptide quantification from degradation experiments, PGP and AcPGP were measured using a Thermo Accela Pump and Autosampler coupled to a Thermo TSQ Quantum Access. HPLC was done using a 2.0 × 150-mm Jupiter 4u Proteo column (Phenomenex, Torrance, CA) with A: 0.1% HCOOH and B: MeCN+0.1% HCOOH: 0-0.5 min 5% buffer B/95% buffer A, then increased over 0.5-2.5 min to 100% buffer B/0% buffer A. Background was removed by flushing with 100% isopropanol/0.1% formic acid. Positive electrospray mass transitions were at 270-70, 270-116 and 270-173 for PGP and 312-140 and 312-112 of AcPGP.

### PGP degradation experiments

Bronchoalveolar lavage fluid (diluted 1/10 in PBS) was incubated with 0.4 mM PGP at 37 °C and 5% CO_2_ for 2 h. Concentrations of PGP remaining were subsequently quantified by electrospray ionization (ESI)–liquid chromatography (LC)/mass spectrometry (MS)/MS (as discussed above) by comparison with PGP standards. The percentage of peptide degraded was determined relative to control samples of 0.4 mM PGP alone.

### Measurement of free proline

Aliquots from PGP degradation experiments were diluted 1 in 10 in PBS (to a final volume of 250 μl). Glacial acetic acid (250 μl) was then added, followed by 250 μl of ninhydrin solution (25 mg/ml in acetic acid/6 M phosphoric acid; heated at 70 °C to dissolve). The reaction mixture was heated at 100 °C for 60 min, allowed to cool to room temperature and the proline containing fraction extracted with 500 μl of toluene and optical density measured at 520 nm.

### LTA_4_H epoxide hydrolase activity

50 μl of the BAL cell suspension was added to 50 μl of RPMI and treated with A23187 at a final concentration of 2 μg/ml. 1% DMSO was used as a control. Cells were incubated for 20 minutes at 37 °C, and supernatants removed and stored at −80 °C for subsequent LTB_4_ analysis (as described below).

### LTB_4_ quantification

The actual concentration of LTB_4_ in the BALF and that generated in the epoxide hydrolase activity assay (above) was assayed using an ELISA, according to manufacturer’s directions (R&D systems) or for maximal sensitivity/specificity using a MDS Sciex (Applied Biosystems) API-4000 spectrometer equipped with a Shimadzu HPLC. HPLC was done using a 2.1 × 100 mm Kinetex column (Phenomenex) with A: 10 mM NH_4_OAc and B: MeCN+10 mM NH_4_OAc: 0–0.5 min 5% buffer B/95% buffer A, then increased over 0.5–5 min to 100% buffer B/0% buffer A. Negative electrospray mass transitions were at 335–195, 335–151 and 335–123 for LTB_4_.

### Cytokine/chemokine quantification

The levels of CXCL1/KC, CXCL2/MIP-2, MCP-1/CCL2, C5a, Lungkine/CXCL15, TNF-α, IL-6 (Duo Set; R&D Systems) and MCP-3/CCL7 (MyBiosource, San Diego, CA) were measured according to the manufacturer’s instructions.

### Albumin ELISA

The concentration of albumin in BALF was measured using an ELISA, according to the manufacturer’s directions (Bethyl Laboratories, Montgomery, TX).

### Lactate dehydrogenase (LDH) assay

LDH activity in BALF was assessed using the Lactate Dehydrogenase Activity Assay Kit (Sigma-Aldrich). Equal volumes of LDH substrate, LDH assay dye and LDH assay cofactor preparation were combined and 100 μl was added to 50 μl of sample. The samples were incubated at room temperature in the dark for 30 min. The reaction was stopped using 30 μl 1 N HCl and absorbance determined using a spectrophotometer (490 nm).

### Real-time PCR

Total RNA was extracted from 50 to 100 mg of lung tissue (azygous lobe) using a Qiagen RNeasy Mini Kit. Total RNA (1 μg) was reverse transcribed into complementary DNA using a High Capacity cDNA Reverse Transcription Kit (Life Technologies) as per the manufacturer’s instructions. Real-time PCR reactions were performed using fast-qPCR mastermix (Life technologies) on a Viaa-7 instrument (Life Technologies) with TaqMan primer sets for murine ENA-78/CXCL5, MCP-1/CCL2, MCP-3/CCL7, MMP-9, MMP-12, GAPDH or HPRT (Life Technologies), and gene expression was analysed using the change-in-threshold ΔΔCt method; fold changes in mRNA expression for targeted genes were calculated relative to naive WT controls.

### Statistical analysis

Statistical significance was calculated using an unpaired Mann–Whitney test. All *P* values of ≤0.05 (*) and ≤0.01 (**) were considered significant and are referred to as such in the text.

## Additional information

**How to cite this article:** Akthar, S. *et al.* Matrikines are key regulators in modulating the amplitude of lung inflammation in acute pulmonary infection. *Nat. Commun.* 6:8423 doi: 10.1038/ncomms9423 (2015).

## Supplementary Material

Supplementary Information Supplementary Figures 1-6

## Figures and Tables

**Table 1:** Characterization of immune cells by flow cytometry.

**Cell type**	**Surface marker phenotype**	**Monoclonal antibody conjugate**	**Catalogue number**	**Dilution**
B cells	CD19^+^CD3^−^	CD19-FITC (BD Biosciences)CD3-PECy7 (eBioscience)	55739825-0031	1/1001/200
Natural killer (NK) cells	NKp46^+^CD3^−^	NKp46-PE (eBioscience)CD3-PECy7 (eBioscience)	12-335125-0031	1/2001/200
CD4^+^ T cells	CD4^+^CD3^+^	CD4-PerCP (BD Biosciences)CD3-PECy7 (eBioscience)	55305225-0031	1/2001/200
CD8^+^ T cells	CD8^+^CD3^+^	CD8-APC (BD Biosciences)CD3-PECy7 (eBioscience)	55303525-0031	1/2001/200
Neutrophils	Ly-6G^high^CD11b^high^CD11c^low^F4/80^low^	Ly6G-FITC (BD Biosciences)CD11b-PerCP (eBioscience)CD11c-APC (BD Biosciences)F4/80-PE (eBioscience)	55146045-011255026112-4801	1/1001/4001/2001/50
Alveolar macrophages	CD11b^low-int.^CD11c^high^F4/80^high^	CD11b-PerCP (eBioscience)CD11c-APC (BD Biosciences)F4/80-PE (eBioscience)	45-011255026112-4801	1/4001/2001/50
Inflammatory monocytes/macrophages	CD11b^high^CD11c^low^F4/80^high^	CD11b-PerCP (eBioscience)CD11c-APC (BD Biosciences)F4/80-PE (eBioscience)	45-011255026112-4801	1/4001/2001/50
