# Supplementary material for: Matrikines are key regulators in modulating the amplitude of lung inflammation in acute pulmonary infection
Source: Nat Commun. 2015 Sep 24;6:8423. doi: 10.1038/ncomms9423 (PMC4595997; doi:10.1038/ncomms9423)
Supplement: Supplementary Information — Supplementary Figures 1-6 [file ncomms9423-s1.pdf]

Matrikines are key regulators in modulating the amplitude of lung inflammation in acute pulmonary infection

Samia Akthar, Dhiren F. Patel, Rebecca C. Beale, Teresa Peiró, Xin Xu, Amit Gaggar, Patricia L. Jackson, J. Edwin Blalock, Clare M. Lloyd, Robert J. Snelgrove.

Supplementary Material.

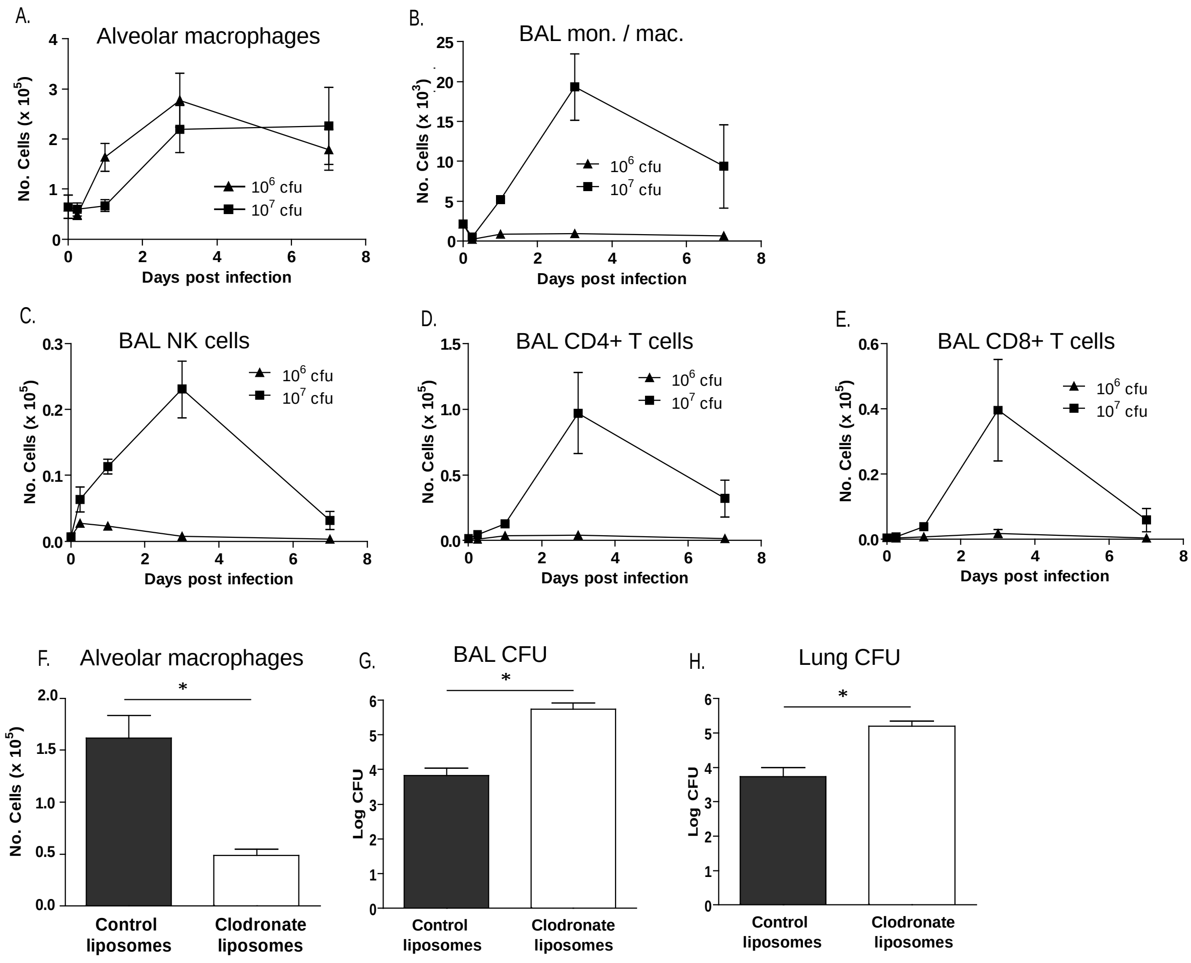

Supplementary figure 1. *Macrophage and lymphocyte response to Haemophilus influenzae b infection.*129/S6 mice were infected intranasally with $1x10^6$ or $1\ x10^7$ *Haemophilus influenzae b* (Hib) and the numbers of alveolar macrophages (**A**), infiltrating monocytes / macrophages (**B**), NK cells (**C**), $CD4^+$ T cells (**D**) and $CD8^+$ T cells (**E**) in the airways of Hib infected mice was determined by flow cytometry. Hib-infected mice were administered control or clodronate liposomes and alveolar macrophage numbers (**F**) and CFU in the airways (**G**) and lung tissue (**H**) assessed at 24 hours post infection. Data (mean ± S.E.M.) are representative of at least 2 experiments with 5-6 mice per group. *, P<0.05; **, P<0.01 using Mann-Whitney.

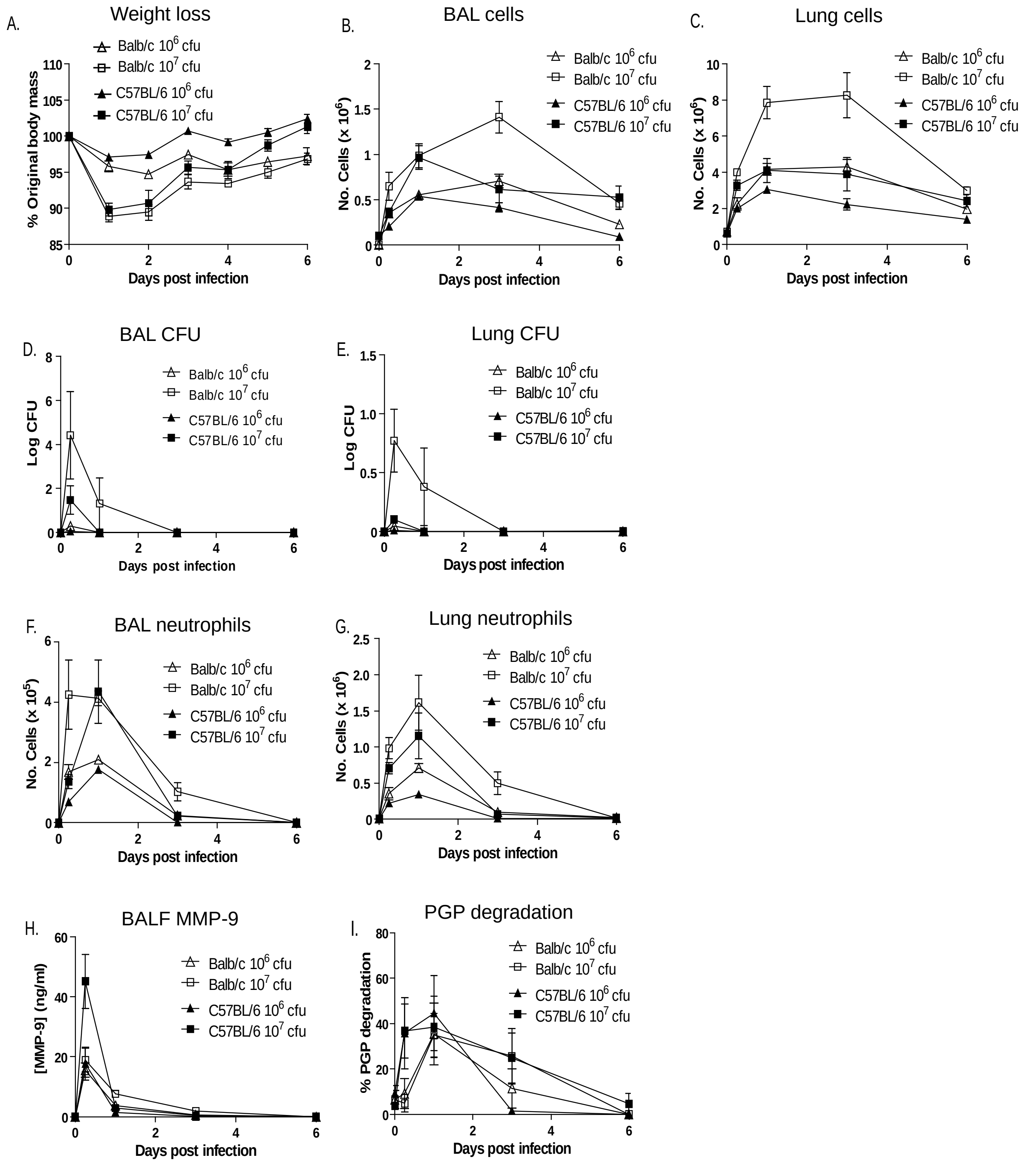

Supplementary figure 2. *Robust, acute pulmonary infiltrate controls Haemophilus influenzae infection in Balb/c and C57BL/6 mice.* Balb/c or C57BL/6 mice were infected intranasally with 1x10^6 or 1 x10^7 *Haemophilus influenzae b* (Hib) and weight loss assessed daily and expressed as a percentage of the original body mass (**A**). Total cell numbers in the airways (**B**) and lung tissue (**C**) of Hib-infected mice was enumerated by trypan blue exclusion. Bacterial burden was

assessed by performing serial dilutions of BALF (**D**) and lung homogenate (**E**) on Brain Heart Infusion (BHI) agar plates and counting colony forming units (CFU). The number of neutrophils recruited into the airways (**F**) and lung tissue (**G**) of Hib infected mice was determined by flow cytometry. Amounts of total MMP-9 were assessed by ELISA in the BALF at different times post infection (**H**). BALF from different time points post-Hib infection, was incubated with 4mM PGP and degradation assessed after 2 hours by mass spectrometry (**I**). Data (mean ± S.E.M.) are representative of at least 2 experiments with 5-6 mice per group. *, $P<0.05$; **, $P<0.01$ using Mann-Whitney.

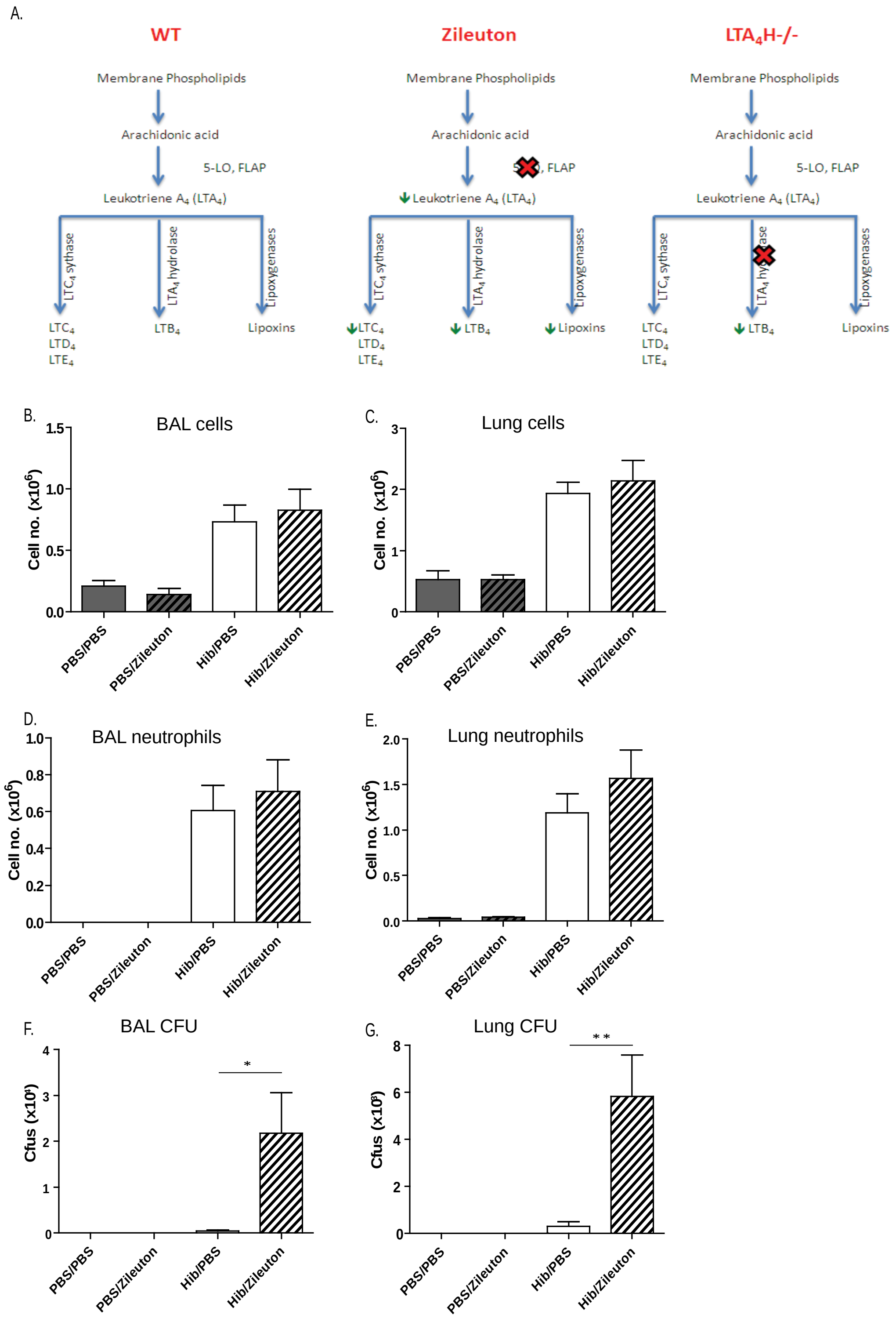

Supplementary figure 3. *5-lipoxygenase inhibition does not alter pulmonary inflammation but compromises bacterial clearance.* (**A**) Arachidonic acid, derived from membrane phospholipids, is converted to leukotriene A_4 (LTA_4) through the action of 5-lipoxygenase (5-LO) and 5-lipoxygenase-activating protein (FLAP). LTA_4 can subsequently be converted to cysteinyl

leukotrienes (LTC_4, D_4, E_4) by the action of LTC_4 synthase, to LTB_4 by the action of leukotriene A_4 hydrolase (LTA_4H) or to lipoxins via the action of lipoxygenases. Zileuton inhibits 5-LO, preventing LTA_4 formation and thus the ensuing generation of cysteinyl leukotrienes, LTB_4 and lipoxins. LTA_4H-/- mice selectively lose the capacity to convert LTA_4 to LTB_4. 129/S6 mice were infected intranasally with 1 $x10^7$ Hib and administered vehicle or zileuton. Total cell numbers in the airways (**B**) and lung tissue (**C**) were enumerated at 24 hours post infection. The number of neutrophils recruited into the airways (**D**) and lung tissue (**E**) of Hib infected mice was determined by flow cytometry. Bacterial burden at this time point was assessed by performing serial dilutions of BALF (**F**) and lung homogenate (**G**) on Brain Heart Infusion (BHI) agar plates. Data (mean ± S.E.M.) are from 5-6 mice per group.*, $P<0.05$; **, $P<0.01$ using Mann-Whitney.

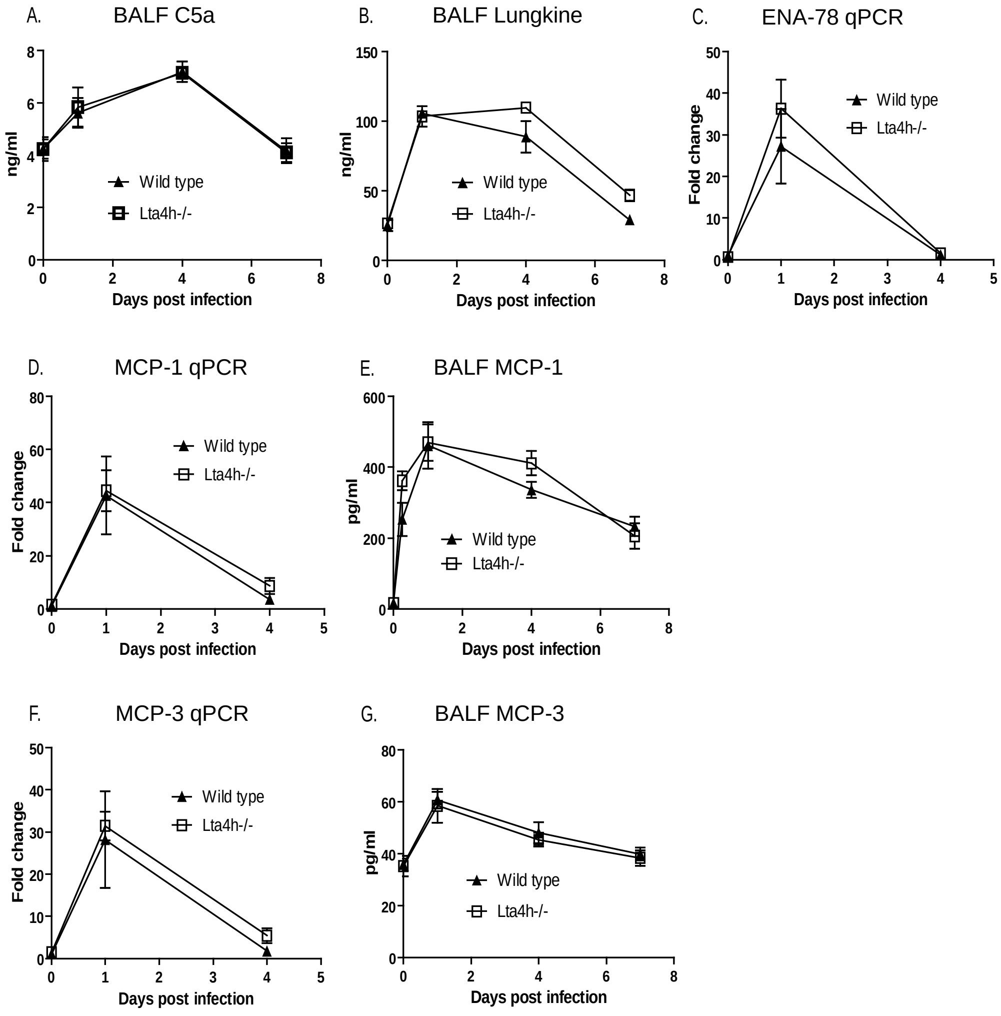

Supplementary figure 4. *Hib-infected lta4h-/- mice display comparable levels of classical neutrophil and monocyte chemoattractants.* Lta4h-/- mice and littermate controls were infected intranasally with $1x10^7$ Hib and levels of C5a (**A**) and lungkine/CXCL15 (**B**) in the BALF were assessed by ELISA. Levels of ENA-78/CXCL5 mRNA were assessed in lung tissue by real time PCR at different times post Hib infection (**C**). Lung mRNA and BALF protein levels of MCP-1/CCL2 (**D** and **E,** respectively) and MCP-3/CCL7 (**F** and **G,** respectively) were determined at different times post Hib infection. Data (mean ± S.E.M.) are representative of at least 2 experiments with 5-6 mice per group (A, B, E and G) or are from a single experiment with 5 mice per group (C, D and F). *, P<0.05; **, P<0.01 using Mann-Whitney.

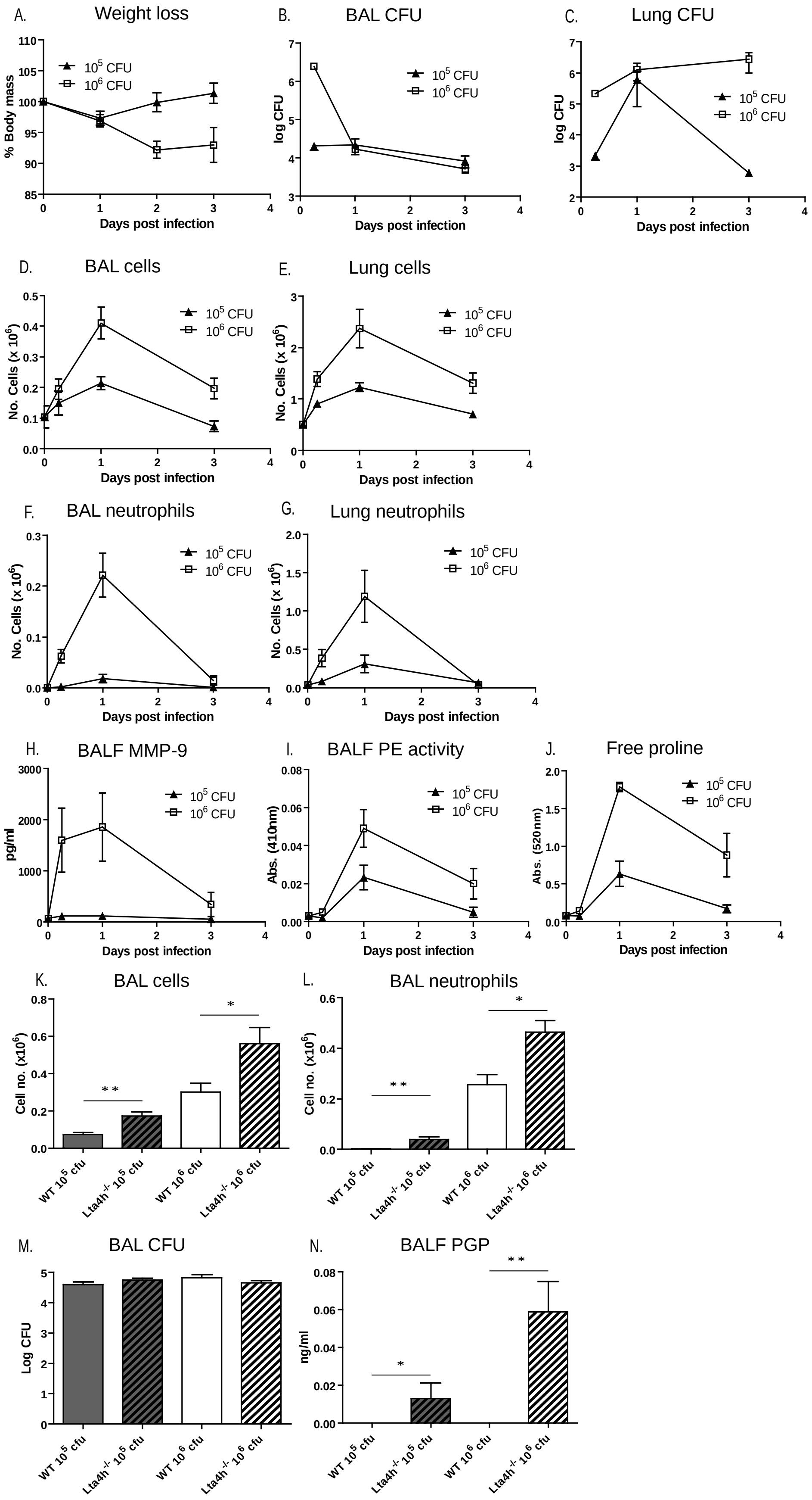
A. Weight loss
% Body mass
Days post infection
10^5 CFU
10^6 CFU
B. BAL CFU
log CFU
C. Lung CFU
D. BAL cells
No. Cells (x 10^6)
E. Lung cells
F. BAL neutrophils
G. Lung neutrophils
H. BALF MMP-9
pg/ml
I. BALF PE activity
Abs. (410nm)
J. Free proline
Abs. (520 nm)
K. BAL cells
Cell no. (x10^6)
WT 10^5 cfu
Lta4h^-/- 10^5 cfu
WT 10^6 cfu
Lta4h^-/- 10^6 cfu
L. BAL neutrophils
M. BAL CFU
Log CFU
N. BALF PGP
ng/ml

Supplementary figure 5. *Streptococcus pneumoniae infected lta4h-/- mice display augmented pulmonary inflammation.* 129/S6 mice were infected intranasally with $1x10^5$ or $1x10^6$ *S. pneumoniae* and weight loss assessed daily and expressed as a percentage of the original body mass (**A**). Bacterial burden was assessed by performing serial dilutions of BALF (**B**) and lung homogenate (**C**) on Columbia blood agar. Total cell numbers in the airways (**D**) and lung tissue (**E**) of *S. pneumoniae* infected mice was assessed. The number of neutrophils recruited into the airways (**F**) and lung tissue (**G**) of *S. pneumoniae* infected mice was determined by flow cytometry. Amounts of total MMP-9 in BALF were assessed by ELISA (**H**) at different times post infection. (**I**) PE activity in BALF at different times after *S. pneumonia* infection was determined by change in absorbance at 410 nm following cleavage of p-nitroaniline from PE specific substrate ZGP-pNA. BALF from different time points post-Hib infection, was incubated with PGP and degradation assessed after 2 hours by release of free proline (**J**). Lta4h-/- mice and littermate controls were infected intranasally with $1x10^5$ or $1\ x10^6$ *S. pneumoniae* and total cell numbers in the airways enumerated at 24 hours post infection (**K**). The number of neutrophils recruited into the airways of *S. pneumoniae* infected mice was determined by flow cytometry (**L**). Bacterial burden was assessed by performing serial dilutions of BALF on Columbia blood agar plates (**M**). The concentration of PGP in BALF was determined by ESI-LC/MS/MS (**N**). Data (mean ± S.E.M.) are from 5-6 mice per group. *, P<0.05; **, P<0.01 using Mann-Whitney.

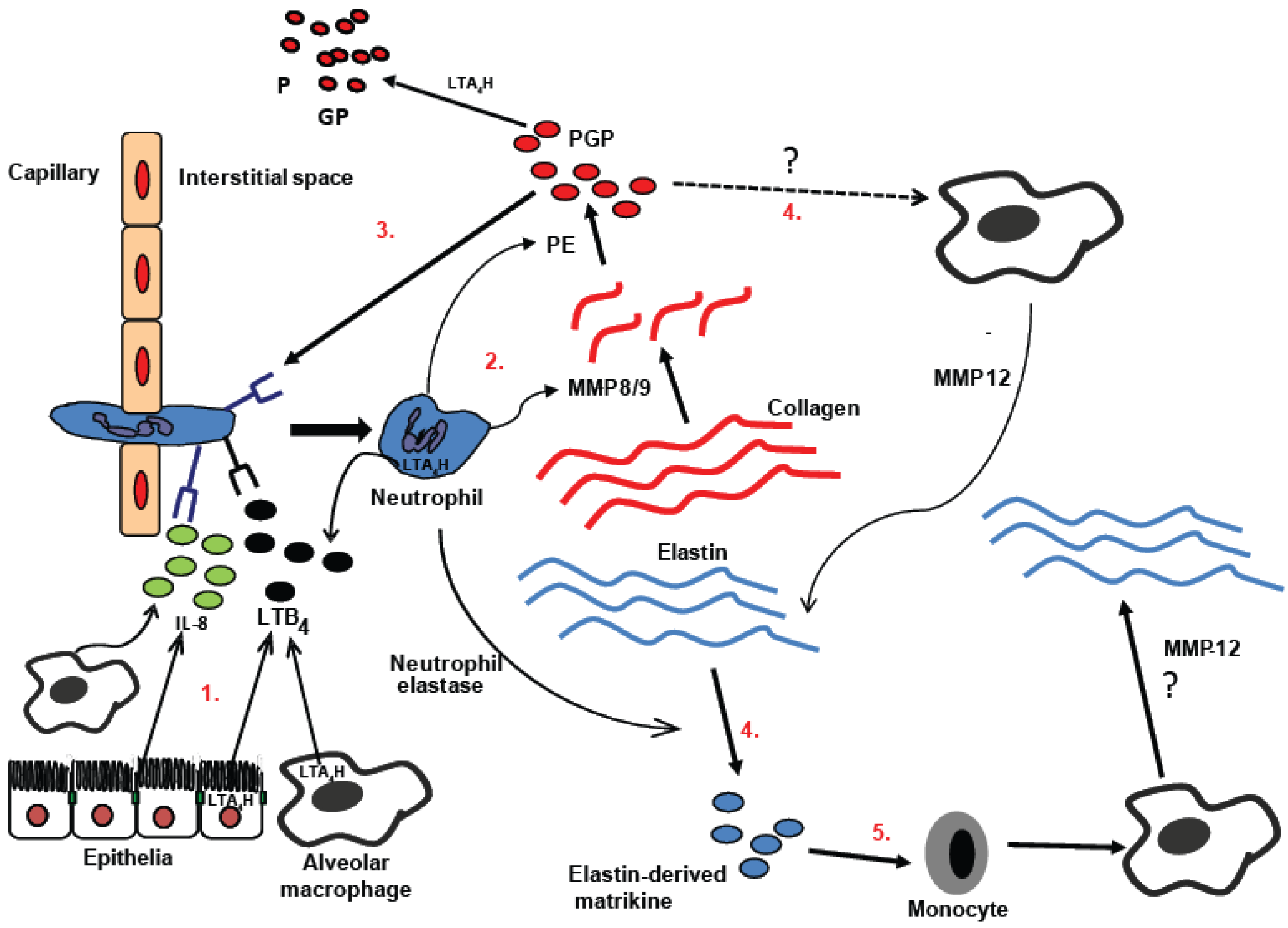

Supplementary figure 6. *A failure to degrade PGP leads to a protease-matrikine discord that exacerbates inflammation.* **1.** In response to infection or injury, pulmonary cells release classical neutrophil chemoattractants, such as IL-8 and LTB_4, leading to the recruitment of neutrophils from the vasculature and into the lung tissue. **2.** Recruited neutrophils release an array of proteases and the concerted actions of MMP-8/9 and PE target extracellular matrix collagen to yield the matrikine PGP. **3.** A failure to degrade PGP by LTA_4H enables it to promote further neutrophil recruitment and release of MMPs/PE with ensuing further generation of PGP – a feed forward mechanism to promote a vicious circle of neutrophilia. **4.** PGP also elicits the release of NE from neutrophils and, indirectly, the release of MMP-12 from alveolar macrophages. These elastases target extracellular matrix elastin to yield elastin-based matrikines that are chemotactic for monocytes. **5.** These chemotactic elastin fragments promote monocyte recruitment. Monocytes mature into tissue macrophages that may produce more MMP-12 to target elastin and generate further matrikines.
